# Al-based functionally graded super-intermetallic compounds for the turbine blade of a high-performance jet engine

**DOI:** 10.1007/s42114-025-01499-4

**Published:** 2025-11-12

**Authors:** Wonjong Jeong, Jeongho Yang, Joon Phil Choi, Ji Yong Hwang, Young Won Kim, Seong Je Park, Jae Won Choi, Woongbeom Heogh, Hoyoung Lee, Jinsoo Park, Min-Kyo Jung, Ji Eun Lee, Ho Jin Ryu, Tae-Sik Jang, Hyun-Do Jung, Mohammad Jahazi, Jubert Pasco, Myoung-Gyu Lee, Hyejin Park, Seung Ki Moon, Rigoberto C. Advincula, Sang Hoon Kim, Clodualdo Aranas

**Affiliations:** 1 Department of Industrial Laser Technology, Korea Institute of Machinery and Materials, Busan, 46744 Republic of Korea; 2 School of Mechanical and Aerospace Engineering, Nanyang Technological University, Singapore, 639798 Singapore; 3 Department of 3D Printing, Korea Institute of Machinery and Materials, Daejeon, 34103 Republic of Korea; 4 School of Mechanical Engineering, Chung-Ang University, Seoul, 06974 Republic of Korea; 5 Industrial Materials Processing R&D Department, Korea Institute of Industrial Technology, Incheon, 21999 Republic of Korea; 6 Korea Additive Manufacturing Innovation Center, Korea Institute of Industrial Technology, Siheung, Gyeonggi-do 15014 Republic of Korea; 7 School of Mechanical Engineering, Gyeongsang National University, Jinju, Gyeongsangnam-do 52828 Republic of Korea; 8 Satellite System 5 Team, Hanwha Systems, Yongin, Gyeonggi-do 17121 Republic of Korea; 9 Department of Materials Science and Engineering & RIAM, Seoul National University, Seoul, 08826 Republic of Korea; 10 Wenzel Marine GmbH & Co. KG, 28816 Stuhr (Brinkum), Germany; 11 Department of Nuclear and Quantum Engineering, Korea Advanced Institute of Science and Technology, Daejeon, 34141 Republic of Korea; 12 Biomedical Convergence Engineering, Pusan National University, Yangsan, Gyeongsangnam-do 50612 Republic of Korea; 13 Department of Bioengineering, Hanyang University, Seoul, 04763 Republic of Korea; 14 Department of Mechanical Engineering, École de Technologie Supérieure, Montreal, Quebec H3C 1K3 Canada; 15 Department of Mechanical Engineering, University of New Brunswick, Fredericton, New Brunswick E3B 5A3 Canada; 16 Department of Mechanical Engineering, Inha University, Incheon, 22212 Republic of Korea; 17 Department of Macromolecular Science and Engineering, Case Western Reserve University, Cleveland, OH 44106 USA; 18 Department of Chemical and Biomolecular Engineering and Joint Institute for Advanced Materials, University of Tennessee, Knoxville, TN 37996 USA; 19 Center for Nanophase Materials and Sciences, Oak Ridge National Laboratory, Oak Ridge, TN 37830 USA; 20 Power Generation Laboratory, Korea Electric Power Research Institute, Daejeon, 34056 Republic of Korea

**Keywords:** Al-based functionally graded structure, Dual-hybrid laser powder bed fusion and directed energy deposition combined with computer numerical control milling, Al-based intermetallic compounds, Turbine blade system, Topological optimization, Precipitation-strengthening super-intermetallic compounds, γ/γ′ microstructures, Functionally graded structure

## Abstract

An Al-based functionally graded structure is fabricated, featuring a discrete compositional gradient of 48.1Al47.9Ti4.0V/73.7Al24.2Ti2.1V/89.5Al10.0Ti0.5V in atomic percentage. This structure is produced via dual-hybrid laser powder bed fusion and directed energy deposition combined with computer numerical control milling. Particularly remarkable is the high tensile strength, ranging from 0.5 to 1.7 GPa. This strength is attributable to three key factors: (1) rapid solidification during inert gas flow following high-energy laser irradiation, (2) the formation of γ-like intermetallic matrix phases along with γ′-like (α_2_-based in the composition of 48.1Al47.9Ti4.0V) intermetallic precipitate phases, and (3) the presence of segregates and precipitates with more V-based compounds at the grain boundaries, distinguishable by their sizes, shapes, and distributions across the microstructures. In addition, (4) large anisotropically lamellar precipitate phases, several hundreds of nanometers in diameter, are predominantly observed in the dendritic regions. Owing to these Al-based intermetallic compounds, each exhibiting low densities (2.9−3.7 g cm^−3^) and high thermal resistances (450−900 °C), the functionally graded structure is then employed in the topological optimization of a turbine blade system for a high-performance jet engine. This process involves identifying the stress-bearing regions, removing any stress-free areas, and applying a structural-stiffness-increasing mechanism through shape and geometric transformation.

**Supplementary Information:**

The online version contains supplementary material available at 10.1007/s42114-025-01499-4.

## Introduction

We introduce a new class of Al-based intermetallic compounds (IMCs) containing Ti and V as secondary and tertiary elements (including TiAl, TiAl_3_, Ti_3_Al, TiAl_2_, Al_10_V, and Al_45_V_7_). Due to their high strengths at elevated temperatures, these IMCs largely retain their functionality at temperatures of up to 650–750 °C, making them potential substitutes for Ni- or Co-based Inconel superalloys [1–4]. Although their specific crystalline structures are different, the nanometer-scale lamellar structures of these novel IMCs are analogous to the γ/γ′ microstructures that are typically observed in precipitation-strengthening superalloys, involving a combination of anisotropically partitioned regions in the matrix phases and irregularly shaped segregates and precipitates at the grain boundaries (GBs) [3, 5, 6]. These lamellar precipitate phases are coherent with the matrix channels of the surrounding TiAl_3_ and Al phases of the respective 73.7Al24.2Ti2.1V and 89.5Al10.0Ti0.5V structures. One notable distinction, however, is the additional formation of nanometer-scale cuboidal precipitates in the Ti- and V-based IMCs, which act to enhance the dispersion strength and grain stabilization [5, 6]. After solid-solution treatment at 1100 °C for 30 min, followed by short-term aging at 480 °C for an additional 30 min to reduce the thermal and residual stresses, the aforementioned microstructures evolve to favor the presence of γ-based matrix phases with fewer γ’-like precipitate phases within the dendrites and grains [7]. The resulting combination of IMCs leads to a tensile yield strength (YS) of 1384 MPa at room temperature, which decreases to 867 MPa at 900 °C. Moreover, while the ductility remains limited (8.2% strain, even at the higher temperature), the plastic deformation response of the functionally graded structure (FGS) can be enhanced by aligning the specific region with an even higher amount of Al to promote the transformation to mainly γ-based Al along with γ′-like TiAl_3_ in the dendritic regions and almost negligible amounts of Al_10_V and Al_45_V_7_ at the GBs [8–10]. This clearly distinguishes the FGS from the conventional cast and wrought alloys, with their uniform crystalline structures [8–10].

From the design perspective, additive manufacturing (AM) facilitates topological optimization (TO) by enabling precise material distribution via the combination of hybrid laser powder bed fusion (LPBF) and directed energy deposition (DED). Thus, with the further application of subtractive manufacturing via computer numerical control (CNC) milling, the fabrication of an FGS is demonstrated for the TO of a turbine blade for a high-performance engine with high tensile strength and thermal resistance (TR), along with low volume and physical density due to its Al-based IMC-containing chemical composition. In particular, the FGS consists of Al-based IMCs with high ultimate tensile strength (UTS) and TR values, and TO is performed after identifying the stress-bearing regions and removing any unnecessary stress-free regions from the core part [11, 12]. Thereafter, we examined how the hierarchical cooling channel (CC), along with the accompanying laser-irradiated, hatching-distance controlled lattice structure, contributes auxiliary mechanical stiffness and less thermal accumulation, thereby enhancing the durability of the supplementary-structure-embedded part [13, 14].

## Experimental methods

The chemical compositions of the powder feedstock and as-additively manufactured structures were analyzed via inductively coupled plasma–optical emission spectroscopy (ICP-OES; Optima 8300, Perkin Elmer, USA). The oxygen and carbon contents were quantified by using an oxygen/nitrogen analyzer (ON-900, Eltra GmbH, Germany) and a carbon/sulfur analyzer (CS-800, Eltra GmbH, Germany), respectively. The particle size distributions were characterized by a laser scattering particle size analyzer (PSA; LS13 320, Beckman Coulter Inc., USA). The powder morphology was observed by scanning electron microscopy (SEM; JSM-5800, JEOL, Japan). Before and after heat treatment (HT), grain orientation mapping of each additively manufactured structure was performed via electron backscatter diffraction (EBSD) on a field-emission SEM (SU70, Hitachi, Japan) equipped with a detector (NordlysNano, Oxford, UK). Selected area electron diffraction (SAED) and energy-dispersive X-ray spectroscopy (EDS) analyses were conducted in transmission electron microscopy (TEM; JEM-ARM200F, JEOL, Japan) to resolve the crystalline structures and local elemental distributions across distinct regions of the FGS. The phase compositions of the powders and as-built samples were further examined by X-ray diffraction (XRD; D/Max-2500VL/PC, Rigaku International Corp., Japan) at 40 kV and 250 mA within a scanning window of 20–80° (2θ). The thermal behaviors of the precursor powders and additively manufactured structures were studied by differential scanning calorimetry (DSC; Q600, TA Instruments, USA) under an Ar flow at a heating rate of 10 K min^−1^. Vickers hardness measurements were conducted on the as-built FGS using a microhardness tester (Duramin-40, Struers, Denmark) under a load of HV_0.5_ in accordance with the American Society for Testing and Materials (ASTM) E92-82 standard. The TS values of specimens produced via LPBF and DED were measured before and after HT on a universal testing machine (Insight-100, MTS Systems, USA) at ambient and elevated temperatures, with a constant crosshead speed of 2.0 mm min^−1^. Further experimental details are provided in the Supplementary Information.

## Results

**Scheme 1:**
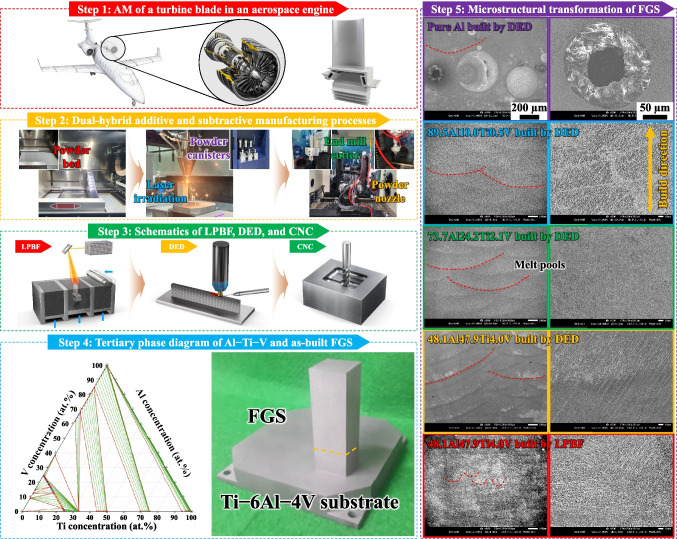
Step 1: Schematic diagrams showing the turbine blade system in a high-performance aerospace engine. Schematic diagrams of the FGS with the discrete compositional ranges of 48.1Al47.9Ti4.0V (by LPBF), 48.1Al47.9Ti4.0V (by DED), 73.7Al24.2Ti2.1V (by DED), 89.5Al10.0Ti0.5V (by DED), and pure Al (by DED). Step 2: Photographs of the dual-hybrid LPBF and DED method combined with CNC milling. Step 3: Schematic diagrams of the dual-hybrid LPBF and DED method combined with CNC milling. Because the bimodal structure with the 45°/90° BD that was obtained when using the sequential LPBF and DED processes had a higher UTS than that of the bimodal structure with either the 0°/90° or 90°/90° BD, the distinctive interfaces existed along the diagonal BD in the middle of the BD-dependent structure. Even though each individual region of the FGS has its own material-strengthening mechanism, the strategic fabrication process enables each specific region to exhibit various TSs in the range of 0.5−1.7 GPa and high TRs in the range of 450−900 °C. Here, the images represent the LPBF-printed 48.1Al47.9Ti4.0V structure and multimodal powder feeding system with the three canisters, which is subjected to the DED process, and the concurrent CNC step. Specifically, the DED-fabricated part of the FGS was built using powders with the chemical compositions of 48.1Al47.9Ti4.0V, 73.7Al24.2Ti2.1V, and 89.5Al10.0Ti0.5V in sequential order. Step 4: The FGS consisting of Al, Ti, and V, based on their tertiary phase diagram. A photograph of the Al-based FGS after surface finishing. Step 5: Microstructures of the pure Al structure built by DED. Each region was indicated in the phase diagram between Al and Ti. Here, the end region of the FGS, which consisted of pure Al built by DED, is seen to contain critical deficiencies such as abundant microstructural defects and mechanical flaws; these were observed irrespective of the applied laser parameters, so that the end region was eventually removed from the FGS

The FGS consisting of 48.1Al47.9Ti4.0V (by LPBF), 48.1Al47.9Ti4.0V (by DED), 73.7Al24.2Ti2.1V (by DED), 89.5Al10.0Ti0.5V (by DED), and pure Al (by DED) along the build direction (BD) is represented schematically in Scheme 1. Initially, the FGS was fabricated using LPBF under an inert Ar atmosphere, then by DED in the middle, and finally by CNC milling for surface refinement (steps 1−3), although some deficiencies occurred due to either insufficient or excessive heat input: the former leads to lack-of-fusion pores, while the latter leads to keyhole-induced cracks [15–17]. Furthermore, the specific regions in the FGS were divided by intersection at 45° along the center of each melt pool when additively manufactured by LPBF to enable building at 90° to the melt pools during the subsequent AM by DED [10, 18, 19]. These specific regions had fewer microstructural defects and mechanical flaws than those built along the 90° direction when using DED as well as those built along the 0° and 90° directions by using LPBF, which were in more contact with the edges of the (energetically less stable) melt pools [18–21]. This is highly applicable to the bimetallic structure, which requires the dual functionality provided by the different BDs and chemical compositions, particularly in terms of the horizontal, diagonal, and vertical orientations, and the proportions of Ti and V as the respective secondary and tertiary elements, according to their as-built positions (step 4). Thus, the various powders (48.1Al47.9Ti4.0V, 73.7Al24.2Ti2.1V, 89.5Al10.0Ti0.5V, and pure Al) were combined in 103 deposition layers to achieve a graded structure starting with 48.1Al47.9Ti4.0V, passing through a series of discrete compositional mixtures with 10 at.% decrements (73.7Al24.2Ti2.1V) and increments (89.5Al10.0Ti0.5V) in the middle, and ending with pure Al. However, the partial structure consisting of pure Al was characterized by a high density of microstructural defects (e.g., voids, dislocations, and stacking faults) and increased mechanical flaws (e.g., pores, cracks, and fractures), which resulted in collapse. Thus, the end region, consisting of only pure Al, was eliminated from the FGS (step 5) for enhanced structural durability and AM producibility. In fact, although 367 layers were built along the FGS to achieve the gradual compositional transition, (1) the thermal and residual stresses at each interfacial region, including that between the initial and second layers, could affect the subsequent interfacial regions due to their close contact [22]. As a result, (2) the as-built FGS could become deformed into an undesired structure with regard to the microstructural abnormalities after being detached from the previous layers during the AM process. However, (3) the mechanical flaws at the interfacial boundaries (IBs) between the dissimilar Al-based layers within the FGS were alleviated because of the gradual transformation induced by the presence of a greater number of Al-based deposition layers, despite the high density of microstructural defects [23]. (4) The number of defects at the IBs between the more numerous Ti-generated layers with high strength but low strain and the fewer Ti-generated layers with low strength but high strain decreased to a greater extent compared to those in the directly bound bimetallic structure, owing to the sudden transformation in the latter. (5) While all of the regions in the FGS solidified into more Al-based phases, the repetition of thermal cycling during continuous laser irradiation (LIr) effectively annealed the entire structure, thereby alleviating the thermal and residual stresses applied during the formation of these regions [24–28]. However, (6) the effects of constitutional supercooling, thermal and residual stress concentrations, and various coefficients of thermal expansion persisted across the microstructures, especially at the 48.1Al47.9Ti4.0V (LPBF)/48.1Al47.9Ti4.0V (DED) interfaces in the FGS, despite the identical chemical composition of each region [29–32].

**Fig. 1:**
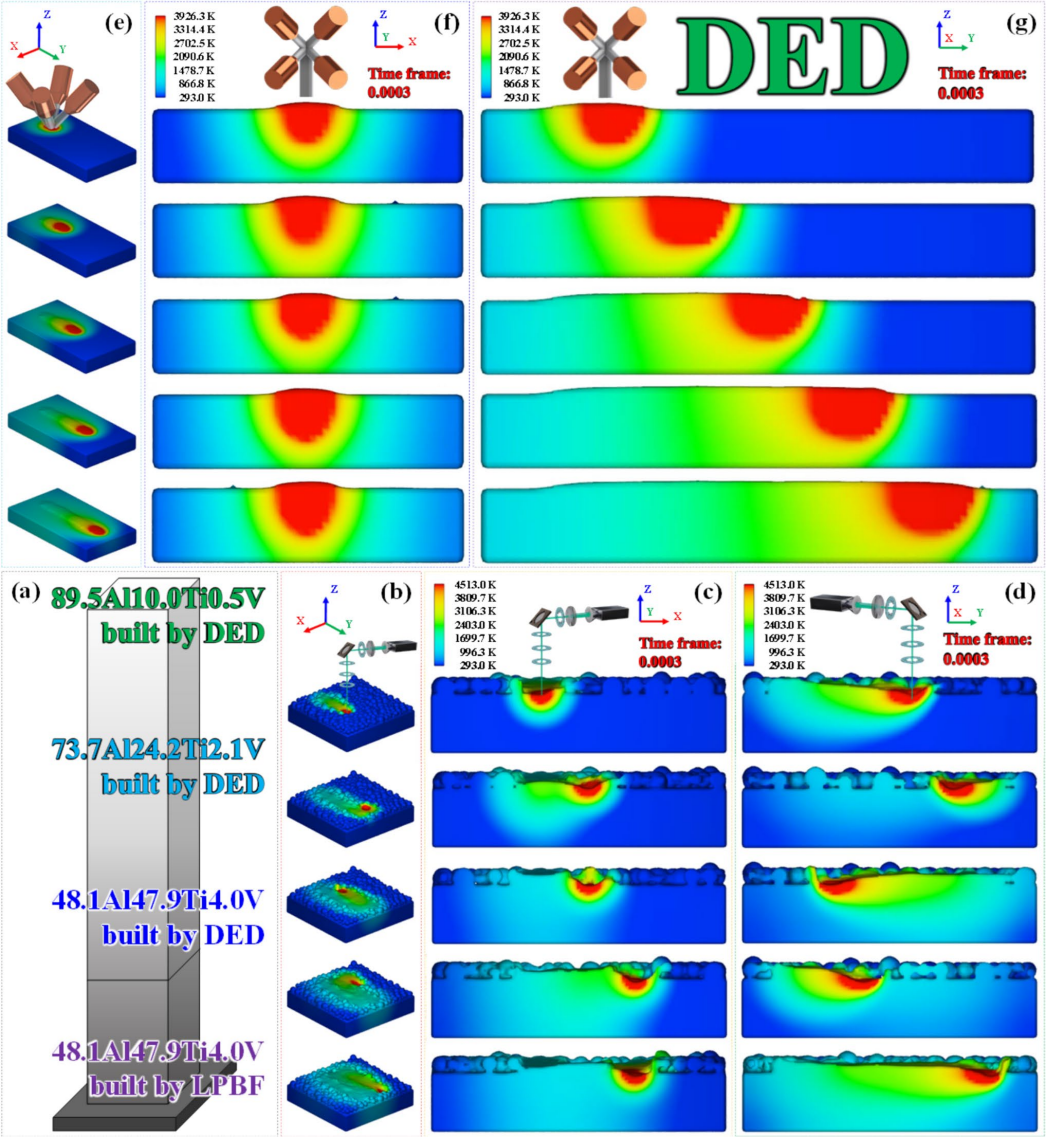
**a**, **b** A 3D view of the triple laser tracks on the 48.1Al47.9Ti4.0V powder bed, along with the temperature gradient field. A constant laser power of 145 W and a scan speed of 16.7 mm min^−1^ produced steady-state thermodynamics of the melt pools in the as-built structure without any microstructural defects and mechanical flaws. **c**, **d** 2D sectional histograms of the triple laser tracks in the above structure, viewed along the horizontal and vertical directions, showing the time-dependent thermal gradient fields. **e** A 3D view of the single laser track on the LPBF-printed 48.1Al47.9Ti4.0V structure with the temperature gradient field when the optimum laser power of 320 W and scan speed of 1000 mm min^−1^ were applied in the DED process. **f**, **g** 2D sectional histograms of the single laser track in the above structure, viewed along the horizontal and vertical directions, showing the time-dependent temperature gradients

The detailed mass-transport, temperature-variation, and fluid-flow profiles are depicted for a single-track melt pool created by application of DED to the triple-track melt pools that were created using LPBF in Figure 1. This specific case is selected in order to save the time required for trial and error, given the extreme difficulty in controlling the parameters of the combined LPBF and DED processes for the Al-based IMCs [33]. Unlike the conventional LPBF process with an almost negligible powder (mass) addition rate, in the DED process, the powder-spraying conditions are generally treated as continuous and uniform in theory [33, 34]. In practice, however, the injected powder particles are discrete and non-uniform [35]. Furthermore, the mass-flow direction on the melt pool surface is outward, which is indicative of a negative surface tension gradient in this region. For example, the temperature of the generated mass is usually lower (the liquidus temperature in this study) than that of the liquid metal on the melt pool surface. Hence, when the powder mass is added to the melt pool, heat exchange occurs, and the temperature decreases. Moreover, owing to the strong mass flow (active powder deposition), the convective heat transfer reduces the temperature of this region. In Figure 1a–d, the mass-flow direction near the top right surface is observed to be downward, which indicates that the spraying powder is just added to this region. As shown in Figure 1e–g, the mass injection decreases the temperature in the top right area. As the distance to the center of the laser spot increases, the temperature decreases. In Figure 1e–g, given that the distance from the laser-beam center increases, a smaller amount of injected powder is deposited. The inward Marangoni flow driven by the surface tension force is dominant [36]. As mentioned previously, we assumed that the powder particles were injected perpendicularly to the melt pools, and that they were at their respective liquidus temperatures. Therefore, these particles can be considered as discrete droplets, and may transform from this discrete phase to a continuous phase on the local melt pool surface, where the powder stream is injected. The force induced by the injected particles was computed as follows:

1 $${F}_{p}=\left[\frac{3\mu {C}_{D}\mathrm{Re}}{4{\rho }_{p} {d}_{p}^{2}} \left({V}_{p} - V\right)+ \frac{g ({\rho }_{p} - \rho )}{{\rho }_{p}}\right]{m}_{p},$$

where *μ* is the viscosity (Pa s), *C*_*D*_ is the drag coefficient, Re is the Reynolds number, *ρ*_*p*_ is the particle density (g m^−3^), *d*_*p*_ is the particle diameter (m), *V*_*p*_ is the particle velocity (m s^−1^), *V* is the velocity (m s^−1^), *g* is the gravitational acceleration (9.8 m s^−2^), *ρ* is the density (g m^−3^), and *m*_*p*_ is the particle mass (g). The drag coefficient can be calculated as follows:

2 $${C}_{D}=\frac{24}{\mathrm{Re}}\mathrm{ for Re}\le 1,$$

3 $${C}_{D}= \frac{24}{\mathrm{Re}} \left(1+0.15\mathrm{ R}{\mathrm{e}}^{0.687}\right)\mathrm{ for }1\le \mathrm{Re}\le 1000,$$

4 $${C}_{D}=0.44\mathrm{ for Re }> 1000,$$

where Re is expressed as follows:

5 $$\mathrm{Re }= \frac{\rho {d}_{p} \left|V - {V}_{p}\right|}{\mu },$$

where the indices are the same as those given in Equation 1. The heat transferred from the injected particles can be computed as follows:

6 $${q}_{p}= {c}_{p} \Delta {T}_{p}{ m}_{p},$$

where *q*_*p*_ is the heat source term (W m^−3^) associated with the powder addition, *c*_*p*_ is the specific heat (J g^−1^ K^−1^), and *T*_*p*_ is the particle temperature (K). Therefore, for the cell computation, the momentum source and heat source terms of the powder injection can be computed as follows:

7 $${p}_{p}=\frac{1}{{V}_{cell}} \sum {F}_{p},$$

8 $${q}_{p} = \frac{1}{{V}_{cell}\Delta t} \sum {Q}_{p},$$

where *p*_*p*_ is the momentum source term (N m^−3^) associated with the particle addition. It is assumed that the powder particles have a uniform size, and that the intensity of the powder stream follows the Gaussian distribution. The powder generation rate can then be defined as follows:

9 $${\dot{N}}_{p}=2 \frac{PFR {\eta }_{p}}{{\overline{m} }_{p} \pi {r}_{p}^{2}} exp \left(-2\frac{{r}^{2}}{{r}_{p}^{2}}\right),$$

where PFR is the powder feeding rate (g s^−1^), *η*_*p*_ is the fraction of powder, ${\overline{m} }_{p}$ is the uniform particle mass (g), *r*_*p*_ is the powder stream radius (m), and *r* is the radius (m) on the *x*-*y* plane. As a result, after reformulation using the cloth simulation filter method, the mass source, momentum source, and heat source terms of the powder flow can be expressed as follows:

10 $$\dot{\mathrm{M}}={\dot{\mathrm{N}}}_{\mathrm{p}}{\overline{\mathrm{m}} }_{\mathrm{p}}\left|\nabla \mathrm{F}\right|\frac{2{\uprho }_{\mathrm{surface}}}{{\uprho }_{\mathrm{m}} + {\uprho }_{\mathrm{g}}},$$

11 $${\mathrm{p}}_{\mathrm{p}}=\left[\frac{3\mu {C}_{D}Re}{{4\overline{\rho }}_{p}{\overline{d} }_{p}^{2} }\left({V}_{p}-V\right)+\frac{g\left({\overline{\rho }}_{p}-\rho \right)}{{\overline{\rho }}_{p}}\right]\Delta \mathrm{t}{\dot{\mathrm{N}}}_{\mathrm{p}}{\overline{\mathrm{m}} }_{\mathrm{p}}\left|\Delta \mathrm{F}\right|\frac{{2\rho }_{\mathrm{surface}}}{{\rho }_{m}+{\rho }_{g}},$$

12 $${\mathrm{q}}_{\mathrm{p}}={\mathrm{c}}_{\mathrm{p}} {\Delta \mathrm{T}}_{\mathrm{p}} {\overline{\mathrm{m}} }_{\mathrm{p}}{\dot{\mathrm{N}}}_{\mathrm{p}}\left|\nabla \mathrm{F}\right|\frac{{2\rho }_{\mathrm{surface}} {\mathrm{c}}_{\mathrm{psurface}}}{{\rho }_{\mathrm{m}}{\mathrm{c}}_{\mathrm{pl}} +{\rho }_{\mathrm{g}} {\mathrm{c}}_{\mathrm{pg}}},$$

where $\dot{M}$ is the mass source term (g s^−1^), *F* is the fluid fraction, *ρ*_surface_ is the surface density (g m^−3^), *ρ*_*m*_ is the liquid density (g m^−3^), *ρ*_*g*_ is the gas density (g m^−3^), ${\overline{\rho }}_{p}$ is the uniform particle density (g m^−3^), ${\overline{d} }_{p}$ is the uniform particle diameter (m), *c*_*p*surface_ is the specific heat (J g^−1^ K^−1^) on the surface, *c*_*pl*_ is the specific heat (J g^−1^ K^−1^) of the liquid, and *c*_*pg*_ is the specific heat (J g^−1^ K^−1^) of the gas.

**Fig. 2:**
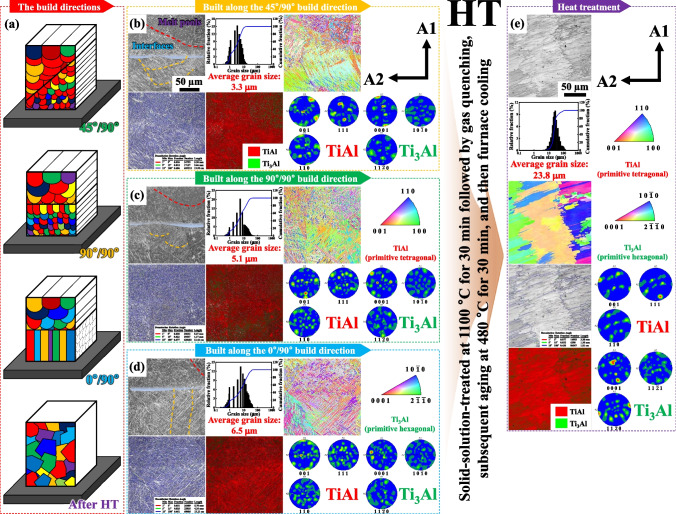
The image quality maps, grain size distributions, and IPF maps of specific regions of the side-plane microstructures at the IBs of the bimodal structures when additively manufactured by the sequential LPBF and DED processes with the BDs of 0°/90°, 45°/90°, and 90°/90° before and after HT. The GB maps (red, < 5°; green, > 5° and < 15°; blue, > 15°), phase maps, and pole figures of the side-plane microstructures at the IBs of the bimodal structures when additively manufactured by the sequential LPBF and DED processes along the BDs of 0°/90°, 45°/90°, and 90°/90° before and after HT. Before HT, hemi-elliptical melt pools were present, with bluntly elongated dendrites and grains located predominantly at their edges, along the direction of heat flow in the diffusive environment during LIr, despite the presence of finely elongated dendrites and grains at the centers of the melt pools. Based on the high density of LAGBs in the bimodal structures, especially at the IBs between the previous (0 0 1) and (1 1 0) planes of the LPBF regions and the following (0 0 1) and (1 1 0) planes of the DED regions, their dissimilarity ratios were smaller for the bimodal structure with the LPBF (along the 45° direction) and DED (along the 90° direction) regions compared to those of the LPBF (along the 0° and 90° directions) and DED (along the 90° direction) regions. After HT, however, the melt pools disappeared, and some bluntly elongated grains were oriented along the identical (0 0 1) and (1 1 0) planes. Furthermore, there were considerably fewer microstructural defects and mechanical flaws in the LPBF and DED regions throughout the microstructures

Before HT, the low-angle grain boundaries (LAGBs), distinct phase constituents, and crystallographic distortions were clearly visible in the contrasting color micrographs (Figure 2) of the bimodal structures fabricated by means of the sequential LPBF (along the 0°, 45°, and 90° directions with the chemical composition of 48.1Al47.9Ti4.0V) and DED (only along the 90° direction but with the same diverse chemical compositions in the FGS consisting of 48.1Al47.9Ti4.0V, 73.7Al24.2Ti2.1V, and 89.5Al10.0Ti0.5V) processes. First, for the bimodal structure built along the 45° direction by using LPBF and the 90° direction by using DED, the regions outlined by the red lines indicated the presence of highly dense LAGBs (5.30 mm in length with an overall length of 148.46 mm) aligned predominantly along the dendrites and grains adjacent to the interfaces. Despite the stronger active diffusion induced by high-energy laser intensity, the densities of microstructural defects and mechanical flaws in the DED regions with a greater number of LAGBs were higher than those in the LPBF regions [20, 27]. The GB maps indicated that the dendrites and grains in the DED regions perpendicular to the direction of LIr were more elongated than those in the LPBF regions perpendicular to the direction of LIr. Furthermore, greater proportions of microstructural defects and mechanical flaws were expected to be aligned with the IBs between the LPBF and DED regions than at the dendrite and grain boundaries within the LPBF and DED regions perpendicular to the direction of LIr [37–39]. However, our adoption of the combined AM process under high-energy LIr for the two powders with the same chemical composition but different powder diameters provided tight bonding between the two additively manufactured phases without any critical microstructural abnormalities (as shown in Figure 2b–d) and mechanical flaws (as shown in Figure S5b−d). In addition, it facilitated identification of the differences between the smaller fan-shaped melt pools formed by LPBF and the larger, ambiguously distinguished melt pools formed by DED (which contributed, to some extent, to the differences between the fraction ratios of the γ-based and γ′-like (α_2_-based) phases in the LPBF and DED regions and the heterogeneous phases in the image quality maps) [10, 40–43]. These were clearly differentiated after identification of the primitive tetragonal crystalline structure of TiAl and the primitive hexagonal crystalline structure of Ti_3_Al in the phase maps [44–46]. In these two built structures, finely elongated dendrites and grains with distinct planes and orientations were seen to spread out from the centers toward the edges of the melt pools in the direction perpendicular to that of LIr, as depicted in the inverse pole figure (IPF) maps. In the case of the 48.1Al47.9Ti4.0 bimodal structure, the grain orientations in both regions (along the 45° direction by LPBF and the 90° direction by DED) were predominantly associated with the same (0 0 1) and (1 0 0) planes, and these mainly consisted of finely oriented dendrites and grains. In the other bimodal regions, however, bluntly elongated dendrites were prevalent to a greater extent in the 0° direction by LPBF and the 90° direction by LPBF than in the 90° direction by DED, especially in the peripheral areas of the α_2_-based phases with more (1 0 $\overline{1 }$ 0) and (1 1 $\overline{2 }$ 1) planes in the IBs. This is because the more energetically stable FGS that was divided by intersection at 45° along the center of each melt pool during LPBF exhibited fewer microstructural defects and mechanical flaws but a larger interfacial surface area than the less energetically stable structures that were built along the 0° and 90° directions by using LPBF in more contact with the edges of the melt pools [21, 47]. This was especially notable at the IBs between the previous (0 0 1) and (1 1 0) planes in the LPBF regions and the identical planes in the DED regions. Consequently, the dissimilarity ratios were the smallest for the FGS with the LPBF (along the 45° direction) and DED (along the 90° direction) regions, which is consistent with the tensile testing results, as discussed in the subsequent section.

**Fig. 3:**
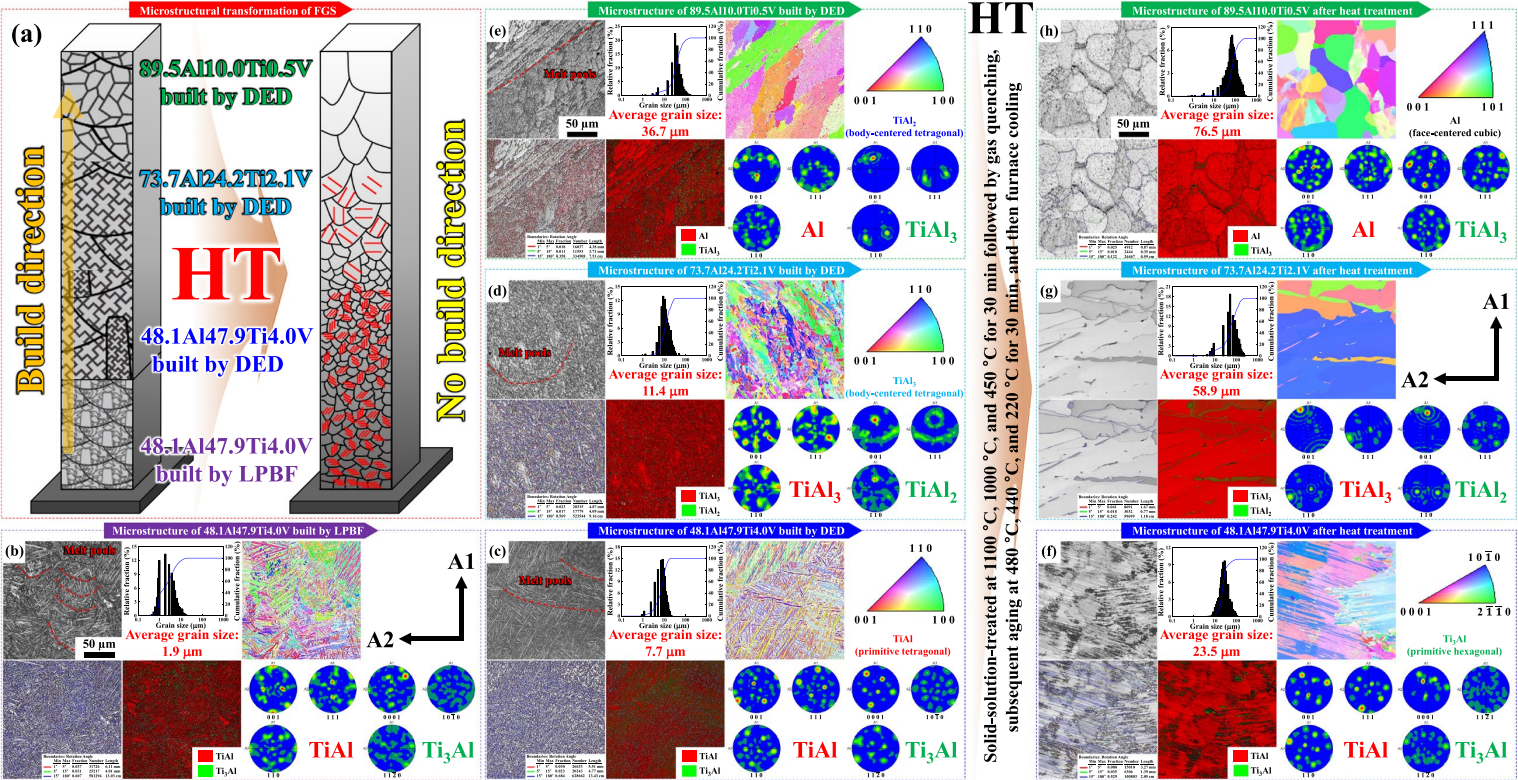
The image quality maps, grain size distributions, and IPF maps of specific regions of the additively manufactured FGS before and after HT, along with the GB maps (red, < 5°; green, > 5° and < 15°; blue, > 15°), phase maps, and pole figures of the γ-like matrix and γ′-like precipitate phases. Before HT, hemi-elliptical melt pools with slightly equiaxial dendrites and grains were present, predominantly at the edges of the melt pools along the direction of heat flow in the diffusive environment during LIr, despite the presence of elongated dendrites and grains at the centers of the melt pools. The secondary precipitate phases were tightly conjugated (entangled) with the primary matrix phases, containing both elongated and equiaxial dendrites and grains perpendicular to the hemi-elliptical melt pools during LIr. After HT, however, the melt pools disappeared, while the remaining dendrites and grains had become almost equiaxial, which were commonly observed in the as-cast microstructure

The as-built melt pools were typically aligned along the dendrite and grain boundaries but more or less differentiated after identifying the γ- and γ′-like phases in the maps of the 73.7Al24.2Ti2.1V and 89.5Al10.0Ti0.5V structures (Figure 3d, e). These features were induced mainly by the formation of primary matrix phases (TiAl_3_ and Al) alongside the secondary precipitate phases (TiAl_2_, TiAl_3_, Al_10_V, and Al_45_V_7_), as observed in the high-magnification microscopy analysis. Subsequently, the as-printed FGS was similarly developed with the elongated dendrites and grains distributed from the center to the edge of each melt pool perpendicular to the direction of LIr in the IPF maps. Considering the similarity to the crystalline structure of each region in the as-printed FGS, the grain orientations of the 73.7Al24.2Ti2.1V and 89.5Al10.0Ti0.5V structures were strongly associated with the preferred (1 1 0) and (1 1 1) planes for the γ-like phases found predominantly at the centers of the melt pools, which were composed primarily of the elongated dendrites and grains [47, 48]. By contrast, in some regions, the equiaxial dendrites and grains with the (1 1 0) and (1 1 1) planes for the γ′-like phases in the edges of the melt pools were predominant, especially in the peripheral areas of the γ-like phases with the (0 0 1) and (1 1 0) planes. However, after HT (solid-solution and subsequent aging), the inhomogeneous microstructures comprising the γ- and γ′-like phases, melt pools, and elongated grains disappeared, and were replaced by the grains with the average sizes of 58.9 and 76.5 μm (Figure 3g, h). The fraction of LAGBs (1° < θ < 5°) associated with the crystallographic distortions found primarily at the dendrite and grain boundaries decreased significantly. The less-preferred textures were present in the IPF maps, and the measured texture-intensity indexes were 2.85 and 1.92, which are indicative of the microstructures with weak textures. In particular, the distributions of microstructural abnormalities and mechanical flaws were considerably sparser, which confirmed the effectiveness of the applied HT in terms of lowering the thermal and residual stresses. Owing to a lack of strong textures, the dendrites and grains exhibited considerably more equiaxial morphologies, and this was accompanied by the transformation of a small number of fine equiaxial grains to larger equiaxial grains in both regions.

**Fig. 4:**
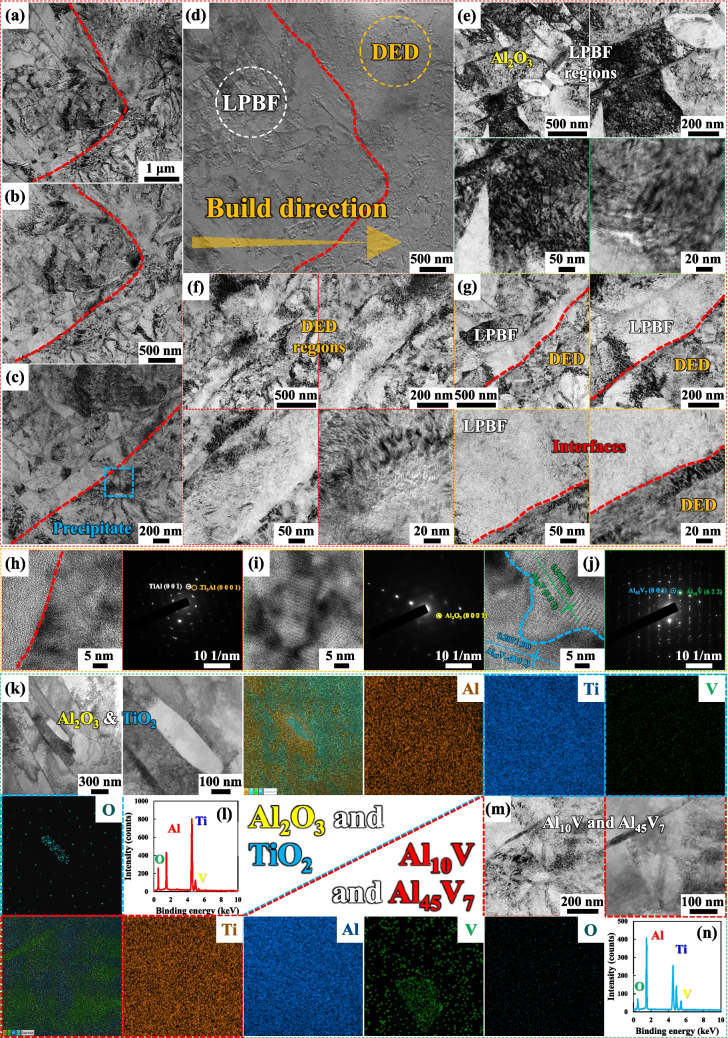
**a**–**d** The TEM images of the primary TiAl matrix phases with the primitive tetragonal crystalline structure alongside the secondary Ti_3_Al precipitate phases with the primitive hexagonal crystalline structure. **b**, **c** The Al_10_V and Al_45_V_7_ compounds with several tens-of-nanometer diameters, which exhibited the face-centered cubic crystalline structure of Al_10_V rather than the end-centered monoclinic crystalline structure of Al_45_V_7_, were more spread out at the IBs, despite being present at the dendrite and grain boundaries and in the hemi-elliptical melt pools in each region of the 48.1Al47.9Ti4.0V bimodal structure. **d** The low-magnification TEM image, **e**–**g** intermediate-magnification TEM images, and **h**–**j** high-magnification TEM images of the primary TiAl matrix and secondary Ti_3_Al precipitate phases, along with the corresponding lattice fringe measurements and SAED patterns. The V-based compounds were formed mostly at the IBs between the LPBF and DED regions rather than at the edges of the interconnected melt pools, while the presence of Al_2_O_3_ and TiO_2_ was revealed to promote oxidation in the LPBF and DED regions during high-energy LIr. However, these oxide phases might have slightly increased the mechanical strength of each region of the bimodal structure through the reinforcement effect. **k**, **l** The HAADF-STEM images, along with the corresponding overall and individual elemental mapping images, and EDS spectrum of the precipitate. According to the elemental counts calculated from the EDS analysis, especially the molecular ratio of Ti to O, this precipitate was composed of TiO_2_. (m, n) The TEM images and corresponding elemental mapping images of the bimodal structure with the γ-based/γ′-like matrix/precipitate phases before HT, revealing that the Ti_3_Al precipitate phases were anisotropically distributed around the TiAl matrix phases, as observed in the SEM analysis, irrespective of the quantities of Al_10_V and Al_45_V_7_, due to their small sizes and low concentrations

Previously, it was mentioned that the FGS was additively manufactured using 48.1Al47.9Ti4.0V (by LPBF), 48.1Al47.9Ti4.0V (by DED), 73.7Al24.2Ti2.1V (by DED), and 89.5Al10Ti0.5V (by DED). However, (1) the morphological features, (2) the microstructural distributions, and (3) the chemical compositions of the segregates and precipitates were ignored in the previous study, and therefore, they are discussed in this section. Furthermore, (4) the TEM images of the unique bimodal structure composed of various Al-based compounds and built along the 45° direction by using LPBF and the 90° direction by using DED are depicted in Figure 4. These images were examined to identify the accumulation of more oxides, IMCs, segregates, and precipitates at the IBs, in addition to their presence at the dendrite and grain boundaries and in the melt pools, with the finely and bluntly columnar precipitate phases in the matrix phases at both the LPBF and DED regions. In detail, the presence of substantial quantities of polygonal Al_2_O_3_, TiO_2_, Al_10_V, and Al_45_V_7_ was highlighted at the distinct IBs owing to the oxidation of Al, Ti, and V, and the formation of V-based IMCs during high-energy LIr. Remarkably, one of the aluminum oxides was embedded near the IMCs, thereby further enhancing the mechanical strength, while reducing the strain and elongation of the bimodal structure through a reinforcement effect compared to that of the singly applied structure [38, 49–51]. Meanwhile, one of the nanoparticles that was aligned explicitly with the specific matrix and precipitate IMCs in Figure 4b represents the other ceramic oxide, namely TiO_2_, which had an effect similar to that of Al_2_O_3_ [52]. In addition, although a few of the segregates and precipitates could rarely be observed, they are distinguishable in Figure 4c. The irregularly shaped, dark-colored IMCs are visible in the intermediate-magnification TEM image in Figure 4g, and the development of the V-based compounds along the interfaces between the different types of dendrites and grains is evident. In addition to the reinforcement effect of the V-based segregates and precipitates, the stabilizing energy of the α_2_-based precipitate phases in the γ-based matrix phases within the LPBF and DED regions ensured that each distinct lamellar microstructure had the same chemical composition [53, 54].

**Fig. 5:**
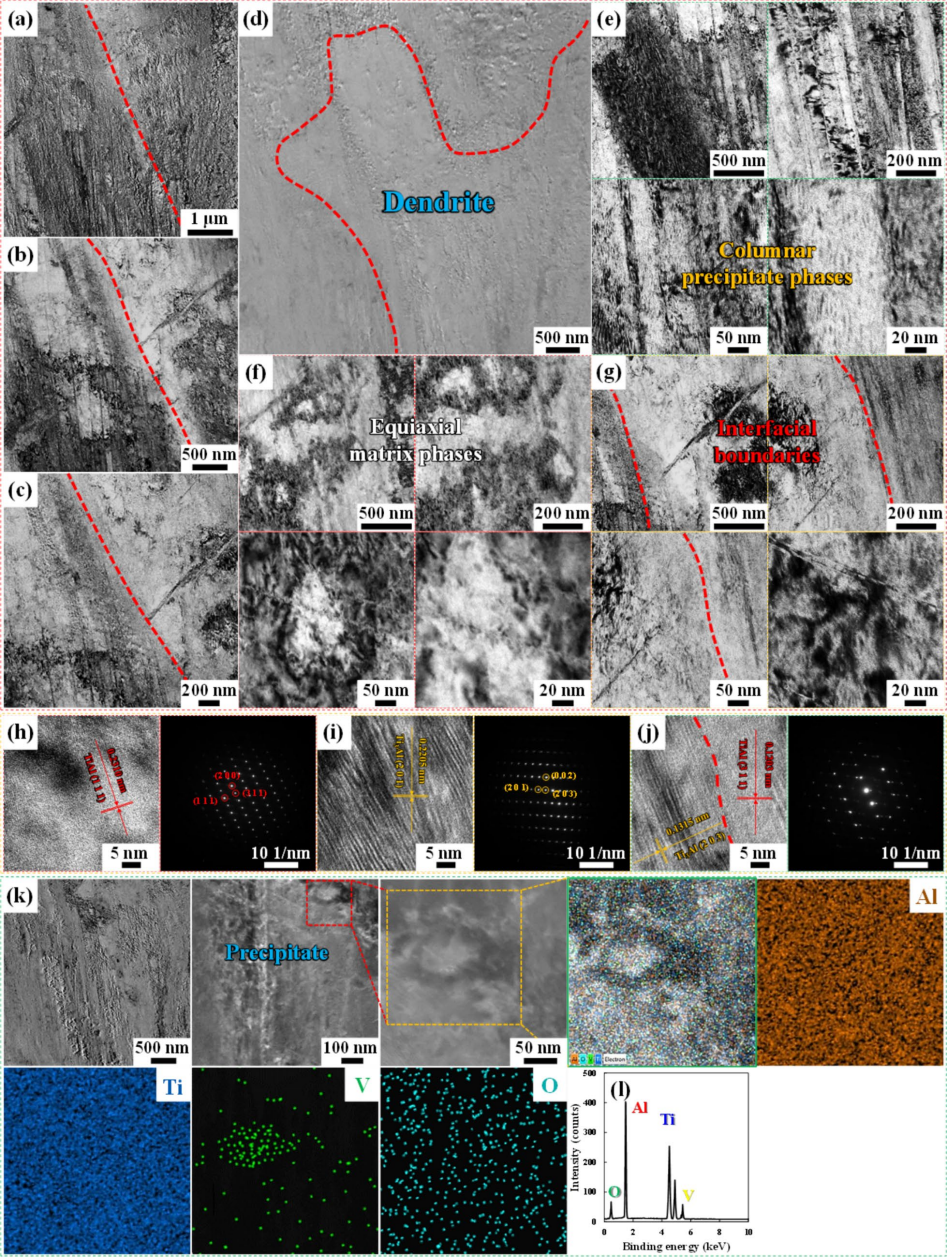
TEM images obtained after HT, showing **a**–**d** the primary TiAl matrix phases and secondary Ti_3_Al precipitate phases and **e**–**g** the Al_10_V and Al_45_V_7_ compounds with the same crystalline structures distributed at the GBs. **d**–**j** The low-magnification TEM image, intermediate-magnification TEM images, and high-resolution TEM images, along with the corresponding lattice fringe measurements and SAED patterns of **f** the primary TiAl matrix and **e** secondary Ti_3_Al precipitate phases. The V-based compounds were formed mostly at the GBs, where they provided dispersion strength and grain stabilization. Although a sufficiently high amount of the secondary Ti_3_Al precipitate phases was evidently present in the primary TiAl matrix phases, there were no additional peaks, which confirmed that no unexpected chemical reactions occurred between the Ti_3_Al and TiAl phases. **k**, **l** The TEM images, HAADF-STEM image, and overall and individual elemental mapping images and EDS spectrum of the FGS, showing the γ-based/γ′-like matrix and precipitate phases. According to the elemental counts (molecular ratios) calculated from the EDS analysis, these precipitates consist of Al_10_V and Al_45_V_7_. Despite their small sizes, low concentrations, and lack of thermal decomposition, the Ti_3_Al precipitate phases are distributed more anisotropically within the TiAl matrix, and the Al_10_V and Al_45_V_7_ compounds are embedded at the distinctive dendrite and grain boundaries

Figure 5 a–d show the low-magnification TEM images of the 48.1Al47.9Ti4.0V bimodal structure after HT. As described above, the larger melt pools (top area) that were built by DED were overlaid on the smaller melt pools (bottom area) that were built by LPBF, and the interfaces between these two types of melt pools were devoid of voids, dislocations, and other defects [29, 55]. This indicates that the formation of strong interfacial bonding was achieved by the high-energy LIr applied herein. However, after HT (solid-solution treatment followed by aging), all of the melt pools had vanished from the top and side planes of the microstructures, leaving more unified α_2_-based Ti_3_Al precipitate phases without any thermal decomposition of the V-based (Al_10_V and Al_45_V_7_) compounds throughout the more anisotropic γ-based TiAl matrix phases. Consequently, a few unified columnar matrix and precipitate phases were observed within the considerably larger equiaxial dendrites and grains that developed throughout the microstructures in the top and side planes. Furthermore, the highly diffusive environment induced tighter bonding between the matrix and precipitate phases [18, 53]. However, despite the application of HT at 1100 °C and subsequent short-term aging at 480 °C for 30 min each, no unexpected compounds emerged in the matrix and precipitate phases, which were similar to those of the 48.1Al47.9Ti4.0V bimodal structure. This is due to the limited interfacial reactions between the TiAl, Ti_3_Al, Al_10_V, and Al_45_V_7_ IMCs, especially between the matrix and precipitate phases. As in the previous study on the as-built bimodal structure, TEM and SAED analyses were performed, along with lattice fringe measurements and EDS mapping scans, to assess the morphological features, heterogeneous distributions, and chemical compositions of the heat-treated matrix and precipitate phases, as well as those of the V-based (Al_10_V and Al_45_V_7_) compounds within the microstructures, as depicted in Figure 5. Notably, the SAED pattern of each specific region near the dendrites and grains revealed the coexistence of overlapping γ-based TiAl and α_2_-based Ti_3_Al phases, alongside the face-centered cubic crystalline structure of Al_10_V and the end-centered monoclinic crystalline structure of Al_45_V_7_. All these patterns conformed to the observed spot and dot patterns, which elucidated the broadly scattered (distributed) small-sized segregates and precipitates adjacent to the other matrix and precipitate phases in the FGS [56–58]. Furthermore, no new peaks emerged between the abundant matrix and lamellar precipitate phases, nor among the segregates and precipitates at the dendrite and grain boundaries, suggesting the absence of unexpected chemical reactions between them under high-energy LIr and subsequent HT. In addition, the high-angle annular dark-field (HAADF) images and corresponding EDS mapping analyses indicated that the matrix phases of TiAl evolved into all of the proposed compounds (including Al_10_V and Al_45_V_7_) rather than into Ti_3_Al precipitate phases. This can be attributed to the high TRs of the various IMC phases, which helped to preserve the intrinsically high deformation resistance provided by the tight lamellar entanglement even at elevated temperatures [26, 27, 59].

**Fig. 6:**
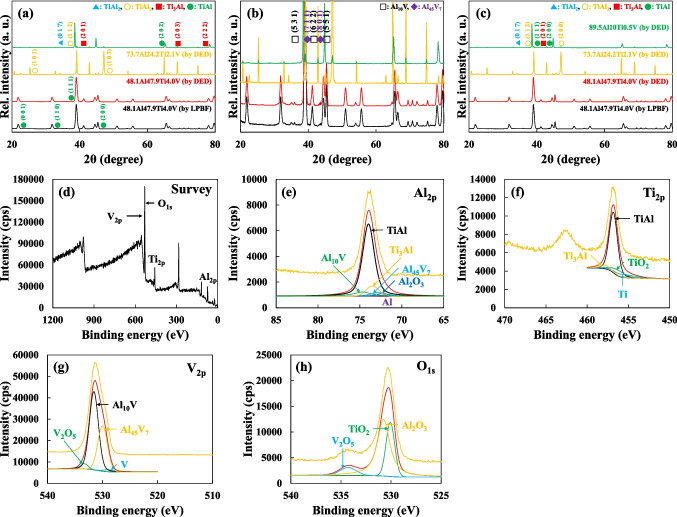
**a**–**c** Although the specific crystalline structures in each functionally graded region differed, the lamellar precipitate IMC phases (i.e., Ti_3_Al, TiAl_2_, and TiAl_3_) were invariably present, along with a combination of anisotropically partitioned regions, within the abundant matrix phases (TiAl, TiAl_3_, and pure Al), while irregularly shaped segregates and precipitates (Al_10_V and Al_45_V_7_) were present at the GBs. Although many more γ′-like precipitate phases were present in the γ-like matrix phases owing to RS during AM, the subsequent HT led to the formation of more homogeneous microstructures in these regions, thus resulting in the formation of a greater number of γ-like matrix phases and fewer γ′-like precipitate phases. **d**–**h** Although the presence of Al_10_V and Al_45_V_7_ was somewhat ambiguous in the XRD analysis results, the XPS analysis results facilitated the precise determination of their compositions

In addition to the preceding panoramic SEM analysis (Figure S7), the crystalline structure of the additively manufactured FGS across each transition range (48.1Al47.9Ti4.0V by DED and 73.7Al24.2Ti2.1V by DED) was further investigated by performing the XRD analysis (Figure 6a–c) to determine which phases were present relative to those in the Al-based IMCs and alloys at each end (48.1Al47.9Ti4.0V by LPBF and 89.5Al10Ti0.5V by DED) [38, 39, 60]. In agreement with the previous results, the initial 48.1Al47.9Ti4.0V structure that was built using LPBF predominantly featured a γ-based TiAl matrix, along with a small amount of γ′-like (α_2_-based) Ti_3_Al precipitates. In the transition range of the 48.1Al47.9Ti4.0V structure that was built using DED, the XRD pattern showed no significant 2θ changes owing to the identical chemical composition and rapid solidification (RS) [61, 62]. This indicated that a unified transition range was maintained in terms of the crystalline structure [63]. Subsequently, as in the previous SAED analysis results, no impurities or other phases were observed in the XRD patterns for both the 73.7Al24.2Ti2.1V and 89.5Al10Ti0.5V structures, which indicated the absence of any unexpected phases over the FGS [38, 64, 65]. The results demonstrated the presence of similar γ-based TiAl_3_ and pure Al matrix phases, along with the distinctive γ′-like TiAl_2_ and other TiAl_3_ precipitate phases. Following the customized HT of each functionally graded region, the variations in peak heights and 2θ positions in the XRD patterns along the FGS from 48.1Al47.9Ti4.0V to 89.5Al10Ti0.5V suggested a certain amount of phase transformation in each transition range. Our analysis of the heat-treated 48.1Al47.9Ti4.0V structure, regardless of whether it was additively manufactured using LPBF or DED, revealed relatively low peak heights for the (2 0 1) and (2 0 3) planes of α_2_-based Ti_3_Al. These low peak heights were correlated with the transformation into considerably fewer lamellar phases over the microstructures. Similarly, the peaks corresponding to the (0 1 7) and (1 1 6) planes of γ′-like TiAl_2_ in the 73.7Al24.2Ti2.1V structure diminished in height owing to the more homogeneous transformation of even a small amount of γ′-like TiAl_2_ into γ-like TiAl_3_, which facilitated the reduction of thermal and residual stresses associated with the heterogeneous phases. For the 89.5Al10Ti0.5V structure after HT, the peak heights of the (1 1 1), (2 0 0), and (2 2 0) planes of the typical γ-based pure Al matrix phases, as well as those of the (1 1 2) and (2 0 0) planes of the γ′-like TiAl_3_ precipitate phases, were almost identical to those of the as-built structure, which clearly indicated that HT had no effect. This was attributed to the complete diffusion of Ti and V at the low melting temperature of pure Al, despite the application of RS after high-energy LIr. Besides, the Al_10_V and Al_45_V_7_ compounds were more precisely characterized by the XPS analysis of the 48.1Al47.9Ti4.0V structure (Figure 6d–h) than by the corresponding XRD analysis. Thus, the various regions in the FGS mostly consist of IMCs with uniform crystalline structures, including similar planes and orientations along the 48.1Al47.9Ti4.0V/73.7Al24.2Ti2.1V/89.5Al10.0Ti0.5V structure, which is useful for the interconnection between each interface, and is consistent with the previous XRD results.

**Fig. 7:**
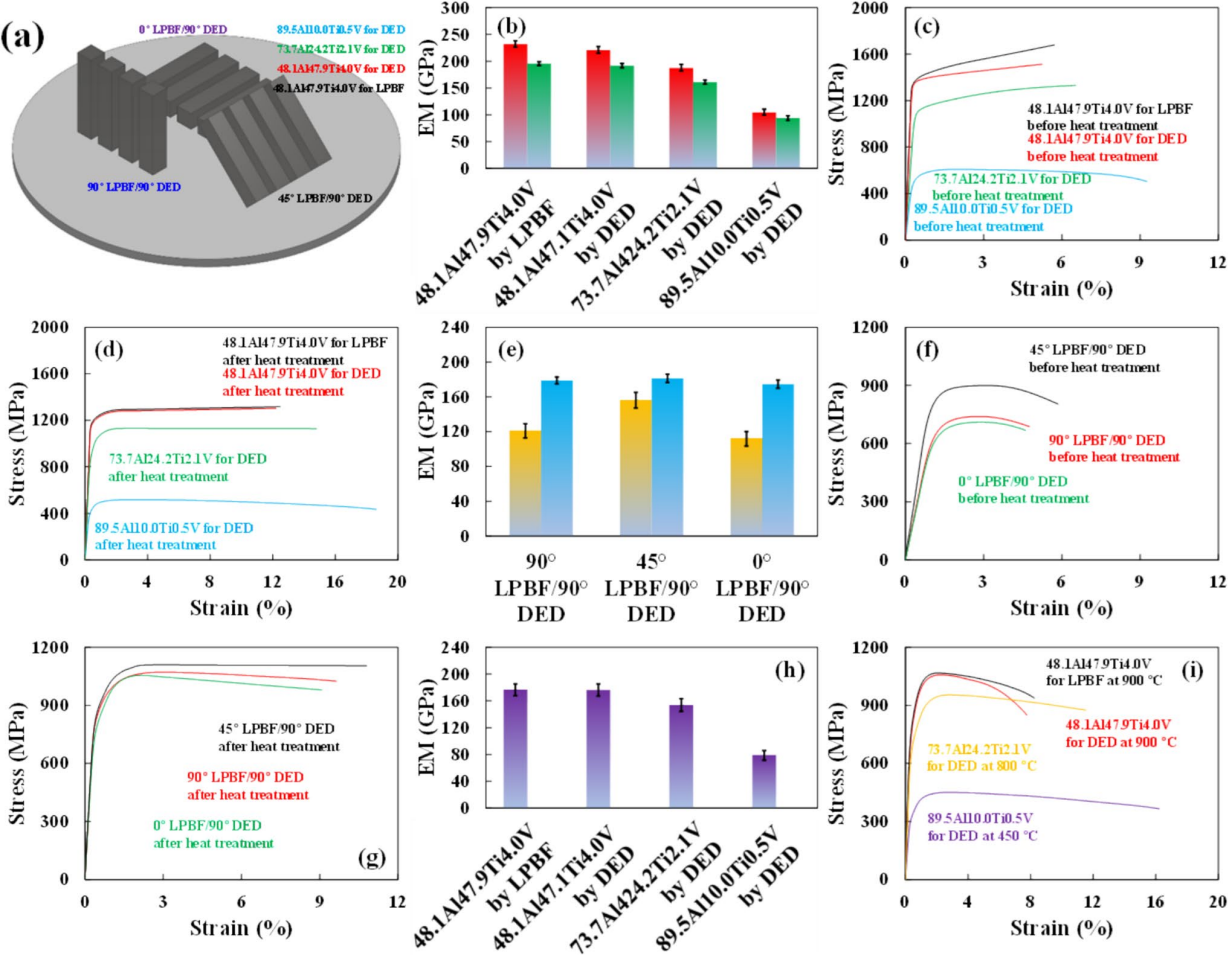
**a**–**c** Before HT, the tensile strains decreased in the order: 89.5Al10.0Ti0.5V built by DED > 73.7Al24.2Ti2.1V built by DED > 48.1Al47.9Ti4.0V built by DED > 48.1Al47.9Ti4.0V built by LPBF, while the TSs correspondingly increased depending on (1) the application of RS, (2) the compositions and concentrations of highly brittle Al-based IMCs, and (3) the presence of more brittle V-based IMCs to increase the dispersion strength and grain stabilization. **e**, **f** Before HT, the bimodal structure with the 45°/90° BD in the sequential LPBF and DED processes allowed a higher strength than those built with the 0°/90° and 90°/90° BDs. **d**, **g**–**i** After HT at high temperatures, however, all the bimodal structures possessed nearly isotropic TSs, regardless of their BDs and despite a few residual microstructural and mechanical deficiencies. The IMC-based 48.1Al47.9Ti4.0V and 73.7Al24.2Ti2.1V structures had abnormally high TRs owing to the compositions of Ti-containing IMCs with high melting temperatures, except for the alloy-based 89.5Al10.0Ti0.5V structure, which consisted mainly of the pure Al phases with the subsidiary TiAl_3_ phases, despite its IMC composition

As shown in Figure 7a–d, a significant decrease was observed in the UTS of each consecutive region of the BD-dependent FGS with the compositional gradient of 48.1Al47.9Ti4.0V, 73.7Al24.2Ti2.1V, and 89.5Al10.0Ti0.5V. This was attributed to the following effects: (1) the formation of more Al-containing IMCs with inherently lower TSs and melting temperatures, (2) the BD effect during AM, (3) the inevitable generation of microstructural defects and mechanical flaws in each layer due to the formation of melt pools regardless of the process used (LPBF or DED), (4) the application of HT to further homogenize the lamellar microstructures, (5) the concomitant alleviation of Hall−Petch strengthening during continuous thermal cycling (annealing), and (6) the distribution of stress during tensile testing at room and high temperatures. Subsequently, the tensile testing results of the bimodal structures, built between the horizontal (0°), diagonal (45°), and vertical (90°) directions by using LPBF and only the 90° direction by using DED but with the identical chemical composition of 48.1Al47.9Ti4.0V, are presented in Figure 7e–g before and after HT. In fact, the primary limitation of employing either LPBF or DED exclusively is that it can lead to an increase in deficiencies due to the longer processing time required and the greater overlap of the powder during AM, especially along the vertical direction [21, 43]. However, employing the bimodal structure with the 45°/90° BD in the sequential LPBF and DED processes resulted in a higher strength than those obtained employing the 0°/90° and 90°/90° BDs. Moreover, in the practical experiment, the DSC−TGA analysis results (Figures S7e and S7f) suggested that the high TRs of the IMC-comprised structures with relatively stronger covalent bonds among Al, Ti, and V compared to those of the alloy-comprised structures were attributable to reduced oxidation at higher scanning temperatures after the formation of thick oxide layers at lower scanning temperatures [66–68]. These findings are consistent with the results of tensile testing at each of various elevated temperatures in our previous study [68, 69].

**Fig. 8:**
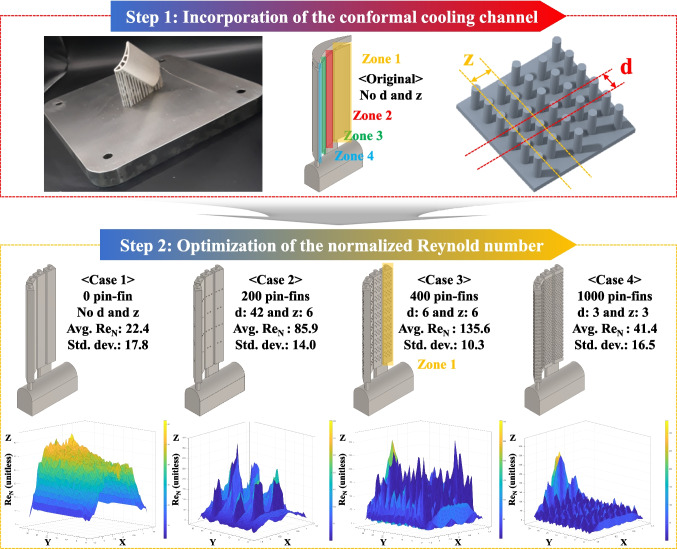
A photographic image of the turbine blade with the conformal CC, along with schematic diagrams showing the AM and TO processes. The pin-fins inside the CC of the turbine blade were designed (arranged) based on the CFD analysis results. The hierarchical CC for each component (airfoil, platform, and root) in the FGS for each region of 48.1Al47.9Ti4.0V and 73.7Al24.2Ti2.1V with a nominal chemical composition was present without any critical deficiencies during AM. A comparative assessment of the varying parameters for cases 1−4 was performed. These values were derived from the Re_N_, with the uniform flow of coolant air being confirmed by CFD simulations. Pin-fin selection was then based on the average Re_N_ and its standard deviation in each case, followed by an evaluation of the CFD outcomes for cases 1−4

We conducted a case study for the TO of a turbine blade as the core part of a high-performance jet engine system in order to demonstrate the practical feasibility of the novel Al-based FGS (Figure 8). Initially, the original turbine blade consisted of a combination of as-cast components, which were subjected to directional or single crystal solidifications. Moreover, the turbine blade featured a complicated aerodynamic airfoil in which each smooth leading edge and sharp-tipped trailing edge was attached to the platform and root in order to sustain a high centrifugal force of 650 N and an intensive hot blast of 1600 °C when in direct contact with the upward flow of cooling air. An intricate arrangement of film cooling holes along the surface of the latter airfoil was designed to draw a cooling curtain in order to protect the main structure from the hot blast, while an internal CC was also provided in order to mitigate any thermal damage due to localized (concentrated) heat accumulation. Furthermore, the internal airfoil, comprising pin-fins, turbulence promoters, and impingement cooling pathways, was directly interconnected to the internal air channel inside the root. For the TO, we analyzed the equivalent stress distributions of force and pressure (load) when the external load interacted with the part at a unique orientation while excluding any structural deformation of the entire structure for compliance minimization (stiffness maximization). Thus, the geometric boundaries were divided according to the TO rule, and the maximum equivalent stresses were found to be one-third of each YS value, i.e., 867 MPa for 48.1Al47.9Ti4.0V by LPBF at 900 °C, 859 MPa for 48.1Al47.9Ti4.0V by DED at 900 °C, and 748 MPa for 73.7Al24.2Ti2.1V by DED at 800 °C. However, the DED process for 89.5Al10.0Ti0.5V and pure Al at each region of the FGS was excluded due to their insufficient TS and TR values based on the previous tensile testing results. Furthermore, the volume reduction was maximized until a safety factor of 3.0 was achieved. From a structural perspective, the newly designed part sufficiently surpassed the required mechanical stiffness and deformation resistance. Inside the topologically optimized turbine blade, a lattice structure was adopted with a hatching distance of 800 µm, as depicted in step 5. As a result, the lattice was topologically optimized for the turbine blade to achieve a safety factor of 3.5, even when incorporating the cellular structure, in order to treat the durability of the jet engine as the top priority. However, although TO of the part can ensure that the turbine blade has the requisite volume while the entire structure has the lowest weight possible, the targeting of each desired region of the FGS-based AM part (especially the airfoil and internal CC) is a complex endeavor. From this geometric perspective, it is difficult to enhance the strengths of the airfoil and directly connected platform components via structural transformations alone. In particular, because the airfoil component requires an even higher resistance to centrifugal force than do the platform and root components, the FGS serves as an auxiliary and effective strengthening tool by imparting the intended strength at each region of specific chemical composition in addition to the BD-dependent strength retention provided by the hybrid LPBF and DED processes, where the diagonal BD results in a higher UTS than the horizontal and vertical BDs. Thus, while gradual material customization occurred during high-energy LIr to result in fewer microstructural defects and mechanical flaws at the IBs, the primary structural design and support structure alignment should be carefully considered when applying the specific material-strengthening mechanism during AM [56, 70, 71].

**Fig. 9:**
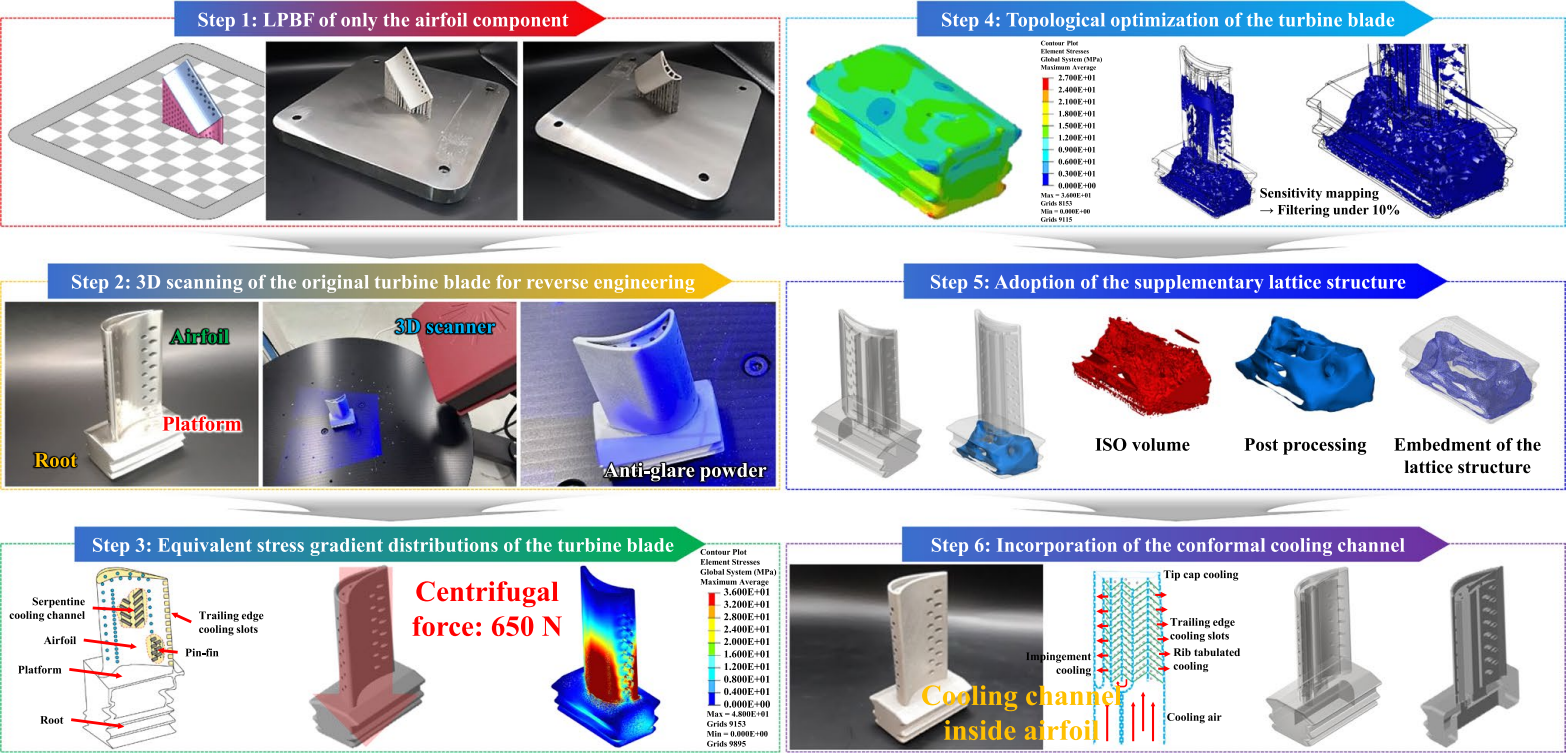
Step 1: Schematic illustrations and photographs presenting the geometry of the turbine blade in the high-performance engine prior to TO. Step 2: The original turbine blade was digitized by 3D scanning, and the corresponding schematic diagrams and photographic images were used to reverse-engineer the geometry. Step 3: The equivalent stress-gradient distributions were evaluated under centrifugal loading and pressure, thereby enabling identification of those regions that require structural retention and those that are suitable for topological reduction before AM. Step 4: TO was conducted with the dual objectives of maximizing the stiffness and minimizing the weight in the BD-dependent region. This region experienced one-third of the maximum equivalent stress (801 MPa) when fabricated along the 45°/90° orientation using sequential LPBF and DED processes, which exhibited a higher strength than the 0°/90° or 90°/90° orientations. The resulting design provided an ergonomically optimized turbine-blade geometry. Step 5: Stress-free zones in the platform and root sections were selectively removed, thereby creating a more intricate wedge-shaped configuration. The mechanical stiffness was further enhanced by integrating an internal lattice structure with an 800 μm hatching distance. Step 6: The pin-fin array within the CC of the airfoil was substantially reconfigured to improve the cooling efficiency

Uniform cooling along a turbine blade is the most critical factor for realizing an aerospace structure with a long lifetime [72]. Thus, the composition of an effective CC with a large surface area as much as possible is the highest priority in the design of the turbine blade [14, 73]. However, the CC should have a high durability with regard to its structural stiffness in order to provide the aerospace part with a high TR against the extensive hot blast [67]. For this reason, the conformal CC should be placed at a uniform depth along the internal surface of the turbine blade [13]. To determine the feasibility of the conformal CC in the turbine blade, an optimized arrangement of pin-fins was designed to induce homogeneous thermal distributions along the conformal CC [74]. Typically, the cylindrical pin-fins are arranged in a specific but broad configuration within the CC, and the quantity of pin-fins directly affects the internal flow field [75, 76]. The design of the surface-type conformal CC inside the turbine blade is divided into four pin-fin zones, where the right component is designated zone 1, and the middle and left components are designated zones 2−4 according to the corresponding coolant-flow pathways (Figure 9). The variables of the parameter study are *d*, which is the distance from the baseline of the even row of pin-fins, and *z*, which is the distance between each pin-fin in the same row. In the pin-fin layout, the parameter *d* defines whether the pin-fins are positioned side-by-side or staggered in a zigzag arrangement, while *z* specifies the number of pin-fins aligned along a row. Following these, *d*_1_ and *z*_1_ are the parameters of case 1, in which the pressure corresponding to the flow rate was the highest. Moreover, *d*_2_ and *z*_2_, *d*_3_ and *z*_3_, and *d*_4_ and *z*_4_ are the parameters of cases 2, 3, and 4, respectively, and the pressures in these cases are considerably lower. Thus, more turbulent air flow was required to effectively achieve the desired cooling efficiency [14, 77]. In addition, the overall coolant flow should be turbulent, not laminar [78]. When the coolant flows slowly, laminar flow generates discrete layers in the flow field, and the resulting surface drag force significantly reduces the flow speed of each adjacent layer from the surface of the CC downward [77, 78]. As a result, the temperature at the end becomes higher than that at the center of the laminar flow [79, 80]. Because the heat flow of the CC is proportional to the temperature difference between the turbine blade and coolant air, the closest boundary layer decreases the heat-transfer efficiency of the coolant air [79, 80]. Therefore, the coolant flow should match the Re, which should neither be too high nor too low.

Accordingly, ANSYS was employed to perform the computational fluid dynamics (CFD) simulation aimed at optimizing the variables d and z under the specified conditions. The governing equations were modeled using the realizable *k*–*ε* model, which consists of two transport equations describing the turbulent kinetic energy (*k*) and its dissipation rate (*ε*). This approach also dynamically estimates the turbulent mixing length (L_T_) and is widely recognized for accurately approximating diverse flow regimes [14, 81]. Compared with the standard *k*–*ε* model, the realizable version offers an improved treatment of flow thermodynamics. Thus, by applying the realizable *k*–*ε* model in the fluid-dynamics simulation, the momentum equation can be solved by substituting the Reynolds stress term, including the eddy viscosity coefficient, into the average time-dependent Navier–Stokes equation:

13 $$\frac{\partial {\overline{U} }_{I}}{\partial {x}_{i}}=0,$$

14 $$\rho \left({\overline{U} }_{j}\frac{\partial {\overline{U} }_{I}}{\partial {x}_{j}}\right) = - \frac{\partial \overline{P}}{\partial {x }_{i}} + \frac{\partial }{\partial {x}_{j}} \left(\mu \frac{\partial {\overline{U} }_{I}}{{\partial x}_{j}} - \rho {u}_{i}{\overline{u} }_{j}\right),$$

where $\overline{U }$_*I*_ denotes the mean velocity (m s^−1^) of the *x*-component, $\overline{U }$_*j*_ denotes the mean velocity (m s^−1^) of the *y*-component, and $\overline{P }$ denotes the mean pressure (Pa). The eddy viscosity coefficient was expressed using the following equation:

15 $${\mu }_{t}= \frac{{C}_{\mu }\rho {k}^{2}}{\varepsilon },$$

where *C*_*μ*_ denotes the coefficient for turbulence viscosity, *k* indicates the turbulence energy (m^2^ s^−2^), and *ε* represents the turbulence energy dissipation rate (m^2^ s^−3^). Unlike the standard *k*–*ε* model, the constant value of *C*_*μ*_ in the realizable *k*–*ε* model is a variable as follows:

16 $${C}_{\mu }= \frac{1}{{A}_{o} + {A}_{S} \frac{kU}{\varepsilon }},$$

where the *A*_0_ and *A*_*S*_ denote constants. The transport equation of the realizable *k*–*ε* model is as follows:

17 $$\frac{\partial }{\partial t} \left(\rho k\right) + \frac{\partial }{\partial {x}_{j}} \left(\rho k{u}_{j}\right) = \frac{\partial }{\partial {x}_{j}} \left[\left(\mu + \frac{{u}_{t}}{{\sigma }_{k}}\right)\frac{\partial k}{\partial {x}_{j}}\right] + {G}_{k} + {G}_{b} - \rho \varepsilon - {Y}_{M} -{ S}_{k},$$

18 $$\frac{\partial}{\partial t}(\rho \varepsilon)+\frac{\partial}{\partial{x_{j}}}(\rho \varepsilon{u_{j}})=\frac{\partial}{\partial{x_{j}}}\left[(\mu + \frac{u_{t}}{\sigma_{k}})\frac{\partial\varepsilon}{\partial{x_{j}}}\right]+ \rho C_{1}S{\varepsilon}-\rho C_{2}\frac{\varepsilon^{2}}{k + \sqrt{v\varepsilon}}+C_{1\varepsilon}\frac{\varepsilon}{k}C_{3\varepsilon}G_{b}+S_{\varepsilon},$$

where *G*_*k*_ represents the turbulence energy (m^2^ s^−2^) generated by the mean velocity, *G*_*b*_ denotes the turbulence energy (m^2^ s^−2^) due to buoyancy, *Y*_*M*_ represents the fluctuation dilation of compressible turbulence in relation to the overall dissipation rate, C_1_, C_2_, C_1ε_, and C_3ε_ denote the coefficients of turbulence viscosity for 1, 2, 1*ε*, and 3*ε*, *σ*_*k*_ and *σ*_*ε*_ are the turbulent Prandtl numbers for *k* and *ε*, and *S*_*k*_ and *S*_*ε*_ are the user-defined source terms, respectively:

19 $${C}_{1}=\mathit{max} \left[0.433, \frac{\eta }{\eta + 5}\right],$$

20 $$\eta = S \frac{k}{\varepsilon },$$

21 $$S=\sqrt{2{S}_{ij}{S}_{ij}},$$

where *S* signifies the modulus of the mean rate of strain tensor. The coefficient of turbulence was used for the default setting value in the ANSYS analysis:

22 $$C_{1\varepsilon}=1.44,C_2=1.9,\sigma_k=1.0,\sigma_\varepsilon=1.2,$$

where the indices are the same as those given in Equations (19) and (21). More detailed information regarding the analysis conditions can be found in Table S4. To compare the CFD analysis results under the same conditions, the velocity of the coolant air at the inlet was fixed at 50 m s^−1^. This made it possible to consider the inlet size and the Re inside the CC of the turbine blade.

As shown in Figure 9, the specific design of the surface-type conformal CC inside the turbine blade leads to the formation of four pin-fin zones [14]. The right component of the CC is designated zone 1, and its middle and left components are designated zones 2–4. In zone 1, where the coolant-flow pressure was relatively high, the normalized Reynolds number (Re_N_) with regard to the flow uniformity and coolant turbulence at the inlet was considered for determining the optimum value for each case depending on the changes in the other parameters [82]. The Re_N_ was determined at the outlet of zone 1, which is indicated by the yellow shadow in Figure 9, and the standard deviation of the Re_N_ was calculated to evaluate flow uniformity and coolant turbulence. Zones 2–4 were located at the middle and left components, respectively, in the CC, and were placed in the main sections that significantly affected the cooling efficiency on the as-additively manufactured part [73]. Subsequently, case 3, in which *d*_1_ = 6 mm and *z*_1_ = 6 mm, had the highest Re_N_, along with the lowest standard deviation thereof, thereby indicating that this was the best value. Cases 1, 2, and 4, in which *d*_1_ = 0 mm and *z*_1_ = 0 mm, *d*_2_ = 42 mm and *z*_2_ = 6 mm, and *d*_4_ = 3 mm and *z*_4_ = 3 mm, respectively, also yielded relatively high Re_N_ values with low standard deviations. The variables and results are confirmed in Tables S4 and S5. As such, using the turbine blade equipped with the conformal CC with the designed pin-fins, most of the areas cooled rapidly and uniformly [14].

## Discussion

In addition to having similar Al- and Ti-based chemical compositions along the 48.1Al47.9Ti4.0V/73.7Al24.2Ti2.1V/89.5Al10.0Ti0.5V structure, each region of the FGS also had a uniform crystalline structure, including similar planes and orientations, which provided tighter bonding between each layer. The initial TiAl matrix compounds exhibiting the (1 1 1) and (2 0 0) planes of the primitive tetragonal crystalline structure were transformed into intermediate TiAl_3_ matrix compounds exhibiting the (1 1 2) and (2 0 0) planes of the body-centered tetragonal crystalline structure, along with other intermediate TiAl_2_ precipitate compounds exhibiting the (0 1 7) and (1 1 6) planes of the body-centered tetragonal crystalline structure. The building process culminated with the final TiAl_3_ precipitate compounds that again exhibited the (1 1 2) and (2 0 0) planes of the body-centered tetragonal crystalline structure. Furthermore, although no impurities or other phases were observed, the microscopic and spectroscopic characteristics of the V-based IMCs did not change significantly across the transition range owing to their small sizes and low concentrations. In the case of the 48.1Al47.9Ti4.0V structure, for example, even when the secondary precipitate phases were tightly intertwined (entangled) with the primary matrix phases along the melt pools with the largest quantities of microstructural abnormalities and mechanical flaws, both in the finely and bluntly elongated dendrites and grains, the network environment extending through the melt pools effectively helped to mitigate the tensile deformation. Nevertheless, while the α_2_-based Ti_3_Al precipitate phases in the γ-based TiAl matrix phases were tightly entangled with each other, which was beneficial for the UTS, the heterogeneous phases were detrimental to the tensile strain. Therefore, delicately controlled post-irradiation HT should be applied to foster more homogeneous microstructures. In terms of the solidification process, our findings suggest that the IBs could have served as favorable segregation and precipitation sites owing to their nucleation-stimulating capability [28, 83]. However, the uneven distributions of these segregates and precipitates at the IBs could impede phase growth after the formation of nucleates [28, 83]. In this way, the inhomogeneous solidification process reduced the deformation resistance of the structure, specifically in terms of tensile strain and elongation, but this decrease may be mitigated by subjecting the highly diffusive bimodal structure to HT [84–86].

With respect to the strength of the FGS, the complementary and synergistic strengthening effects of the Ti-based precipitate phases in the γ-like matrix phases, along with those of the other V-based segregates and precipitates at the dendrite and grain boundaries, were nullified by the transformation from 48.1Al47.9Ti4.0V to 89.5Al10.0Ti0.5V. As the amount of Al in each region of the FGS increased, the benefit of a TS increment disappeared owing to a corresponding decrease in the amounts of highly brittle Ti-containing IMCs [87]. However, the process of alloying with pure Al (for the 89.5Al10.0Ti0.5V structure), which has a high tensile strain, increased the elongation to 9.2% before HT and to 18.6% after HT at the elevated temperature of 450 °C. Furthermore, HT enabled all the structures to exhibit nearly isotropic TSs based on only the chemical compositions, regardless of the application of the LPBF and DED processes. This was the case, even though the microstructural defects and mechanical flaws were reduced, but could not be eliminated completely, even after active solid-solution treatment followed by aging [88]. Nevertheless, the strains were significantly increased when the microstructural defects and mechanical flaws were reduced below a critical extent [53, 54]. To reduce both the defects and flaws at many, however, more advanced post-treatments, such as hot isostatic pressing, cold isostatic pressing, forging, and spark plasma sintering, are required before or during HT [89–91].

For practical application, we propose a turbine blade with excellent TR at each region of the FGS, including an effective conformal CC [92]. The dual-hybrid LPBF and DED method combined with the CNC milling system is used to ensure effectiveness, along with superior accuracy and precision, in the production of the complex CC in the as-designed FGS-based turbine blade, which cannot be manufactured using conventional casting and milling techniques [93].

## Conclusions

The high UTS (0.5–1.7 GPa) and TR (450–900 °C) of the BD-dependent Al-based FGS can be attributed to RS during inert gas flow, followed by LIr, and the formation of various IMCs (primarily TiAl, TiAl_3_, and pure Al, along with subsidiary Ti_3_Al, TiAl_2_, TiAl_3_, Al_10_V, and Al_45_V_7_). In addition to the low density (within the range of 2.9–3.7 g cm^−3^) of the FGS owing to its Al-based chemical composition, the proposed dual-hybrid LPBF and DED method combined with CNC milling induced a specific material-strengthening mechanism at each targeted region during the construction of the turbine blade, which requires a comprehensively high dimensional accuracy and precision to ensure smooth and fluent thermodynamics. From a structural standpoint, the supplementary lattice structure and conformal CC in the turbine blade offered auxiliary mechanical stiffness and less thermal accumulation, thereby improving the durability of the additional-structure-embedded part throughout the combined layer-by-layer additive and subtractive manufacturing processes. Utilizing this approach, the specific complex part was fabricated according to the principles of advanced manufacturing to evaluate the feasibility of practically (or even commercially) implementing the turbine blade system in a high-performance aerospace engine.

## Supplementary Information

Below is the link to the electronic supplementary material.

Supplementary file1 (DOCX 25.6 MB)

## Data Availability

No datasets were generated or analysed during the current study.
