# Supplementary material for: Al-based functionally graded super-intermetallic compounds for the turbine blade of a high-performance jet engine
Source: Adv Compos Hybrid Mater. 2025 Nov 12;8(6):420. doi: 10.1007/s42114-025-01499-4 (PMC12612025; doi:10.1007/s42114-025-01499-4)
Supplement: Supplementary file 1 — Supplementary file1 (DOCX 25.6 MB) [file 42114_2025_1499_MOESM1_ESM.docx]

**Supplementary Information**

**Al-based functionally graded super-intermetallic compounds for the turbine blade of a high-performance jet engine**

Wonjong Jeong^1,a^, Jeongho Yang^2,6,a^, Joon Phil Choi^3,4,a^, Ji Yong Hwang^5^, Young Won Kim^6^, Seong Je Park^7^, Jae Won Choi^6^, Woongbeom Heogh^8^, Hoyoung Lee^9^, Jinsoo Park^10^, Min-Kyo Jung^3^, Ji Eun Lee^6^, Ho Jin Ryu^11^, Tae-Sik Jang^12^, Hyun-Do Jung^13^, Mohammad Jahazi^14^, Jubert Pasco^15^, Myoung-Gyu Lee^9^, Hyejin Park^16^, Seung Ki Moon^2^, Rigoberto C. Advincula^17,18,19^, Sang Hoon Kim^20,^*, Clodualdo Aranas Jr.^15,^*

^1^ Department of Industrial Laser Technology, Korea Institute of Machinery and Materials, Busan 46744, Republic of Korea

^2^ School of Mechanical and Aerospace Engineering, Nanyang Technological University, Singapore 639798, Singapore

^3^ Department of 3D Printing, Korea Institute of Machinery and Materials, Daejeon 34103, Republic of Korea

^4^ School of Mechanical Engineering, Chung-Ang University, Seoul 06974, Republic of Korea

^5^ Industrial Materials Processing R&D Department, Korea Institute of Industrial Technology, Incheon 21999, Republic of Korea

^6^ Korea Additive Manufacturing Innovation Center, Korea Institute of Industrial Technology, Siheung, Gyeonggi-do 15014, Republic of Korea

^7^ School of Mechanical Engineering, Gyeongsang National University, Jinju, Gyeongsangnam-do 52828, Republic of Korea

^8^ Satellite System 5 Team, Hanwha Systems, Yongin, Gyeonggi-do 17121, Republic of Korea

^9^ Department of Materials Science and Engineering & RIAM, Seoul National University, Seoul 08826, Republic of Korea

^10^ Wenzel Marine GmbH & Co. KG, Stuhr (Brinkum) 28816, Germany

^11^ Department of Nuclear and Quantum Engineering, Korea Advanced Institute of Science and Technology, Daejeon 34141, Republic of Korea

^12^ Biomedical Convergence Engineering, Pusan National University, Yangsan, Gyeongsangnam-do 50612, Republic of Korea

^13^ Department of Bioengineering, Hanyang University, Seoul 04763, Republic of Korea

^14^ Department of Mechanical Engineering, École de Technologie Supérieure, Montreal, Quebec H3C 1K3, Canada

^15^ Department of Mechanical Engineering, University of New Brunswick, Fredericton, New Brunswick E3B 5A3, Canada

^16^ Department of Mechanical Engineering, Inha University, Incheon 22212, Republic of Korea

^17^ Department of Macromolecular Science and Engineering, Case Western Reserve University, Cleveland, Ohio 44106, USA

^18^ Department of Chemical and Biomolecular Engineering and Joint Institute for Advanced Materials, University of Tennessee, Knoxville, Tennessee 37996, USA

^19^ Center for Nanophase Materials and Sciences, Oak Ridge National Laboratory, Oak Ridge, Tennessee 37830, USA

^20^ Power Generation Laboratory, Korea Electric Power Research Institute, Daejeon 34056, Republic of Korea

^a^ Wonjong Jeong, Jeongho Yang, and Joon Phil Choi contributed equally to this work.

* Corresponding authors:

E-mail: sanghooni791@naver.com (Sang Hoon Kim).

E-mail: clod.aranas@unb.ca (Clodualdo Aranas Jr.).

**Fabrication of the 48.1Al47.9Ti4.0V (for laser powder bed fusion), 48.1Al47.9Ti4.0V (for directed energy deposition), 73.7Al24.2Ti2.1V (for directed energy deposition), and 89.5Al10.0Ti0.5V (for directed energy deposition) powders**

As shown in **Figures S1**a and **S1**b, the gas-atomized 48.1Al47.9Ti4.0V micropowder displayed slightly crumpled yet spherical particles, making it suitable for the LPBF process [1, 2]. A few satellite particles are seen to be attached to the surface of the core powder [2, 3]. Due to the sudden temperature reduction, the smaller, more fluid droplets (particles) consolidated on the larger, more slowly cooling powder to form a spherical core/shell structure [2, 3]. The powder exhibited a smooth surface because nucleate formation and phase growth were impossible during RS, even though the powder was heat treated to relieve the thermal and residual stresses on the surface [2, 3]. As demonstrated in **Figure S1**c, the PSA revealed a broad size distribution with a median diameter of 36 μm for the 48.1Al47.9Ti4.0V micropowder, aligning with the SEM observations. EDS was performed across the entire area to evaluate the chemical composition of the powder (**Figure S1**d). The highlighted area showed a homogeneous composition throughout each scanned powder, containing the primary elements Al and Ti, along with additional elements such as V and O, and minor amounts of N and H. Although HT was applied to further relieve the thermal and residual stresses on the surface of the 48.1Al47.9Ti4.0V powder owing to the RS process, the powder still maintained its primitive tetragonal and hexagonal crystalline structures, as shown in **Figure S1**e. In the present study, a gas atomizer (Electrode Induction Melting Inert Gas Atomization System, ALD Vacuum Technologies GmbH, Netherlands) was employed to fabricate the 48.1Al47.9Ti4.0V powder from an ingot. For the gas atomization process, 4 kg of the 48.1Al47.9Ti4.0V ingot was melted by heating up to 1800 °C. The molten 48.1Al47.9Ti4.0V liquid was then atomized to form small particles, which rapidly solidified under a gas flow of 30 bar. The collected 48.1Al47.9Ti4.0V powder was passed through a series of standard sieves (ASTM E11) to obtain the desired particle size range of 10−45 μm. The classified 48.1Al47.9Ti4.0V powder was heat treated at 480 °C for 30 min in a vacuum oven for further surface consolidation, and the obtained powder was then subjected to LPBF. Using identical methods and techniques, 48.1Al47.9Ti4.0V, 73.7Al24.2Ti2.1V, 89.5Al10.0Ti0.5V, and pure Al powders (**Figures S1** and **S2**) with less spherical and larger particles (a diameter range of 50−150 μm) were fabricated, analyzed, and used in the DED process.

**Additive manufacturing of the functionally graded structure from laser powder bed fusion of 48.1Al47.9Ti4.0V to directed energy deposition of 89.5Al10.0Ti0.5V**

Powders of 48.1Al47.9Ti4.0V, 73.7Al24.2Ti2.1V, 89.5Al10.0Ti0.5V, and pure Al for use in LPBF (DMP flex 350, 3D Systems, USA) and DED (LENS™, OPTOMEC, USA) were prepared by gas atomization [1, 4]. Initially, the 48.1Al47.9Ti4.0V structure was additively manufactured using LPBF with a layer thickness of 30 μm by applying a laser intensity of 145 W, a hatching distance of 82 μm, and a laser speed of 16.7 mm min^−1^ (**Table S1**). Next, 3−4 laser scans were applied to the LPBF surface in order to alleviate the residual stresses, microstructural defects, and mechanical flaws. After that, the DED-based AM process was used with a laser intensity of 320 W, a spot size of 1 mm, a laser speed of 1000 mm min^–1^, and an Ar carrier-gas flow rate of 45.5 L min^–1^ in order to fabricate a layer with the same chemical composition but having a thickness of 400 μm. Thus, the same chemical composition of 48.1Al47.9Ti4.0V was observed both immediately after LPBF and after the subsequent DED. After that, the DED process was continued with these same laser parameters (except for a reduced laser intensity of 125 W) in order to build the 73.7Al24.2Ti2.1V structure. By contrast, the fourth 89.5Al10.0Ti0.5V structure was built using a substantially lower laser intensity of 55 W, along with a layer thickness of 400 μm, a spot size of 1 mm, a laser speed of 2050 mm min^−1^, and an Ar flow rate of 45.5 L min^−1^. Finally, we attempted to fabricate the pure Al structure by DED; however, this AM process was unsuccessful due to the emergence of abundant microstructural defects and mechanical flaws, regardless of the applied laser parameters [5−7]. All the build rates for these structures varied from approximately 5−125 cm^3^ h^−1^, dependent on the structural composition. A specific layer orientation (245° rotation per layer) was employed during LIr to build the FGS on a Ti−6Al−4V substrate measuring 250 mm in diameter and 15 mm in thickness. Each intermediate (transitional) structure was additively manufactured using the optimized laser parameters under an Ar atmosphere. Subsequently, the resulting FGS was machined using a subsidiary CNC milling machine (Hi−TECH 700, Hwacheon Machinery Co., Ltd., Korea) combined with the DED printer for subtractive manufacturing [8, 9]. The subtractive manufacturing process is divided into separate roughing and finishing processes due to the different machining parameters required for these operations. Under roughing, there are two operations, namely: (i) 3-axis rotation for the model area and (ii) 5-axis rotation for the blade area. Under finishing, the operations are blade finishing and hub finishing, respectively. Different tool diameters were used depending on the operation as follows: 16-mm-flat-end mill for model area clearance (3-axis), 8-mm-ball-nose for blade area clearance (5-axis), and 6-mm-ball-nose for both blade finishing and hub finishing. For model area clearance (3-axis), the feed rate, spindle speed, depth of cut, and stepover are 175 mm min^−1^, 1150 rpm, 1 mm, and 12 mm, respectively. For blade area clearance (5-axis), the corresponding values are 800 mm min^−1^, 2000 rpm, 0.5 mm, and 0.8 mm, respectively. For both blade finishing and hub finishing, the values are 1200 mm min^−1^, 3000 rpm, 0.2 mm, and 0.2 mm, respectively. For the complete integration of these various manufacturing systems, we combined the LPBF-based AM technique with the hybrid DED-based AM and CNC milling techniques through a semi-automatic conveyor belt connected with pulleys under an inert Ar gas atmosphere. Then, the 48.1Al47.9Ti4.0V, 73.7Al24.2Ti2.1V, and 89.5Al10.0Ti0.5V structures, irrespective of LPBF or DED used to fabricate them, were solid-solution-treated at 1100, 1000, and 450 °C for 30 min each, followed by rapid cooling to 25 °C within 10 min by means of gas quenching. These cooled structures were subsequently aged at 480, 440, and 220 °C for 30 min each, and then slowly cooled to 25 °C in a furnace to (1) alleviate the thermal and residual stresses generated during LIr and those accumulated from gas quenching during solid-solution treatment, (2) reduce the numbers of microstructural defects and mechanical flaws, and (3) transform the heterogeneous phases into more homogeneous microstructures in the abundance of IMCs [6, 10, 11]. The resulting lightweight, low-density structures (**Table S2**), which were fabricated by various AM techniques and methods, were present to compare our super-IMCs with the references in terms of mechanical strength and TR. Selection was based on the specific requirements of various target applications and transportation systems.

**Governing equations of numerical simulations at each Al-based region in the functionally graded structure**

The heat-transfer and fluid-convection histograms of the 48.1Al47.9Ti4.0V, 73.7Al24.2Ti2.1V, 89.5Al10.0Ti0.5V, and pure Al structures, additively manufactured via LPBF and DED with either the optimized or default laser-control parameters, as a function of duration were simulated using commercially available fluid dynamics analysis software (FLOW−3D, Flow Science Inc., USA) [12−14]. The governing equations, including the conservation of mass, momentum, volume fraction, and thermal energy, for the AM process of the as-built structures, irrespective of the application of LPBF or DED, are expressed as follows:

$\frac{\text{∂}\rho}{\text{∂}t} + \nabla\cdot\left( \rho V \right) = \bar{M}$, (1)

$\frac{\text{∂}}{\text{∂t}}\left( \rho V \right) + \nabla\left( \rho VV \right) = -\nabla P + \nabla\mu\left( \nabla V + \nabla V^{T} \right) + p_{b} + p_{s}$, (2)

$\frac{\text{∂F}}{\text{∂t}} + \nabla\cdot\left( \mathrm{VF} \right) = \bar{F}$, (3)

$\frac{\text{∂}}{\text{∂t}}\left( \rho H \right) + \nabla\cdot\left( \rho VH \right) = \nabla\cdot\left( k\nabla T \right) + q_{\mathrm{pt}} + q_{l} + q_{\mathrm{loss}}$, (4)

where ρ is the density (g m^−3^), V is the velocity (m s^−1^), $\bar{M}$ is the mass source term (g s^−1^), P is the pressure (Pa), µ is the viscosity (Pa s), p_b_ is the momentum (N m^−3^) induced by buoyancy force, p_s_ is the surface-tension-induced momentum (N m^−3^), F is the dimensionless fluid fraction, $\bar{F}$ is the fluid fraction change rate (s^−1^), H is the enthalpy (J g^−1^), k is the thermal conductivity (W m^−1^ K^−1^), q_pt_ is the phase-transformation-induced heat source/sink term (W m^−3^), q_l_ is the laser power source term (W m^−3^), and q_loss_ is the heat loss term (W m^−3^) by convection and radiation. The fluid-volume method was used to track the fluid-free surface, and the scalar factor F was employed to refer to the fluid fraction in the mesh cell [14]. For a specific fluid in a mesh cell, F = 0 indicates that the cell contains no fluid, whereas F = 1 indicates that the cell is entirely occupied by fluid [14]. A mesh cell with an F value between 0 and 1 suggests a mixture with a specific amount of fluid [14]. Meanwhile, the liquid-volume fraction is determined as follows:

$f_{l} = \left\{ \begin{aligned} 0 (T \leq T_{s}) \\ \frac{T - T_{s}}{T_{l} - T_{s}} (T_{s} < T < T_{l}) \\ 1 (T \geq T_{l}) \end{aligned} \right.$, (5)

where T is the temperature (K) applied to the structure, T_s_ is the solidus temperature (K), and T_l_ is the liquidus temperature (K). Thereafter, the damping force is introduced to decrease the migration velocity of the liquid phase in the mushy zone during the phase transformation, which is described as follows:

$p_{\mathrm{pt}} = -A_{\mathrm{mushy}} \frac{\left( 1 - f_{l}^{2} \right)}{\left( f_{l}^{3} + \delta_{0} \right)} V$, (6)

where A_mushy_ is the constant of the mushy zone (K m^−3^ s^−1^) and $\delta_{0}$ is a small constant to avoid division by zero. The enthalpy of the structure in the solid−liquid mushy zone can be calculated using **Equation (7)** as follows:

$\Delta H_{\mathrm{pt}} = H_{\mathrm{ref}} + \int_{T_{\mathrm{ref}}}^{T} c_{p}\mathrm{dT} + f_{l}L$, (7)

where H_ref_ is the reference enthalpy (J g^−1^) and c_p_ is the specific heat (J g^−1^ K^−1^). Then, the phase-transformation-induced heat source/sink term can be calculated as follows:

$q_{\mathrm{pt}} = - \frac{\text{∂}}{\text{∂t}} \left( \rho\Delta H_{\mathrm{pt}} \right) - \nabla\cdot(\rho V\Delta H_{\mathrm{pt}})$, (8)

where the indices are the same as those given in **Equations (1)**−**(7)**. When high-energy LIr is applied to the functionally graded regions that were additively manufactured by LPBF and DED, the laser heat flux in the numerical analysis area can be described as follows:

$q = \frac{2\eta Q}{\pi r_{s}^{2}} \exp(- \frac{2w^{2}}{r_{s}^{2}})$, (9)

where q denotes the heat flux (W m^−3^), η denotes the absorbance of the structure, Q symbolizes the laser power (W), w indicates the radial distance (m) from the beam center, and r_s_ denotes the laser-spot radius (m). The given heat loss term from the free surface is as follows:

$q_{\mathrm{loss}} = h_{c} \left( T - T_{a} \right) + \varepsilon\sigma\left( T^{4} - T_{a}^{4} \right) + q_{\mathrm{evp}}$, (10)

where h_c_ is the convective heat transfer coefficient (W m^−2^ K^−1^), T_a_ is the ambient temperature (K), ε is the dimensionless emissivity, σ is the Stefan−Boltzmann constant (W m^−2^ K^−4^), and q_evp_ is the heat of evaporation (J g^−1^). Assuming a heterogeneous mixture of metal vapor for simplicity, the heat of evaporation is given as follows:

$q_{\mathrm{evp}} = \frac{0.01\Delta H_{v}^{*}}{\sqrt{2\pi MRT}}P_{0} exp (\frac{\Delta H_{v}^{*} (T - T_{v})}{\mathrm{RT}T_{v}})$, (11)

where $H_{v}^{*}$ is the effective enthalpy (J mol^−1^) of the metal vapor, M is the molar mass (g mol^−1^), R is the universal gas constant (8.314 J mol^−1^ K^−1^), P_0_ is the atmospheric pressure (Pa), and T_v_ is the boiling temperature (K). Above the evaporation temperature, the recoil pressure is generated in both regions, additively manufactured by LPBF and DED, on any free surface of the FGS and is expressed as follows:

$P_{\mathrm{recoil}} = 0.54 P_{0} exp (\frac{\Delta H_{v} (T - T_{v})}{\mathrm{RT}T_{v}})$, (12)

where the indices are the same as those given in **Equation (11)**. In addition, the Marangoni shear stress is induced by the spatial variation in the surface tension force:

$\gamma= \gamma_{m} + \frac{d\gamma}{\mathrm{dT}} \Delta T$, (13)

where γ is the surface tension (N m^−1^) at the surface temperature and γ_m_ is the surface tension (g s^−2^) at the melting temperature. The body force applied in each melt pool includes the buoyancy, gravity, and phase-transformation-induced forces [15]. The buoyancy force is induced due to the temperature-dependent density of the bulk structure in the melt pools [15]. The convective flow caused by the buoyancy force is in the opposite direction to the force of gravity [15]. The momentum source term for buoyancy and gravity is expressed as follows:

$p_{\mathrm{bg}} = \rho g - \rho_{l}\beta_{l} \left( T - T_{L} \right) g$, (14)

where p_bg_ is the momentum (N m^−3^) induced by buoyancy and gravity, g is the gravitational acceleration (9.8 m s^−2^), ρ_l_ is the liquid density (g m^−3^), and β_l_ is the coefficient of thermal expansion (K^−1^). The temperature-dependent density is calculated as follows:

$\rho= \rho_{l} - \rho_{l}\beta_{l} \left( T - T_{L} \right)$, (15)

where the indices are the same as those given in **Equation (14)**. The temperature gradient is in the direction normal to the solid−liquid interface in the solidification front, and can be expressed as follows:

$G = (\frac{\partial T}{\partial x}, \frac{\partial T}{\partial y}, \frac{\partial T}{\partial z})$, (16)

where G is the temperature gradient (K m^−1^). The solidification rate is calculated as follows:

$R_{s}= TS\cdot\cos\theta_{s}$, (17)

where TS is the travel speed (m s^−1^). As this model uses the Cartesian coordinate system, the direction of LIr corresponds to the positive x-axis direction. The value of cos θ_s_ can be rewritten as follows:

$\cos\theta_{s}= \frac{\partial T}{\partial x}\cdot\frac{1}{\left| G \right|}$, (18)

where the indices are the same as those given in **Equations (15)**−**(17)**. The heat loss term of the melt pools includes the convective, radiation, and evaporation heat loss terms. The convective and radiation heat loss terms can be written as follows:

$q_{c}+ q_{r} = - \left[ h_{c} \left( T - T_{0} \right) + \sigma\varepsilon\left( T^{4}- T_{0}^{4} \right) \right] \left| \nabla F \right| \frac{2\rho_{\mathrm{surface}}c_{\mathrm{psurface}}}{\rho_{l}c_{\mathrm{pl}} + \rho_{g}c_{\mathrm{pg}}}$, (19)

where q_c_ is the convective heat loss term (W m^−3^), q_r_ is the radiation heat loss term (W m^−3^), T_0_ is the environment temperature (K), ρ_surface_ is the density (g m^−3^) of the interface, c_psurface_ is the specific heat (J g^−1^ K^−1^) on the surface, ρ_m_ is the interface liquid density (g m^−3^), c_pl_ is the specific heat (J g^−1^ K^−1^) of the liquid, ρ_g_ is the interface gas density (g m^−3^), and c_pg_ is the specific heat (J g^−1^ K^−1^) of the gas. The evaporation heat loss term is defined as follows:

$q_{\mathrm{evp}}= - 0.01 \frac{L_{\mathrm{evp}}M}{\sqrt{2\pi MRT}} P_{0} exp [\frac{{\Delta H}_{\mathrm{evp}} (T - T_{\mathrm{evp}})}{\mathrm{RTT}_{\mathrm{evp}}}] \left| \nabla F \right| \frac{2\rho_{\mathrm{surface}}c_{\mathrm{psurface}}}{\rho_{m}c_{\mathrm{pm}} + \rho_{g}c_{\mathrm{pg}}}$, (20)

where L_evp_ is the evaporation latent heat (J g^−1^), H_evp_ is the evaporation enthalpy (J g^−1^), and T_evp_ is the evaporation temperature (K). The thermo-physical properties of pure Al are listed, along with other variables, in **Table S3**. As micron-sized powder particles are used in the present study, the influence of the Gibbs−Thomson effect on the melting temperature was ignored [12−14]. As illustrated in **Figures 2** and **S3**, to increase the calculation efficiency, a partial volume was selected as the calculation domain in this model [14]. A user-defined subroutine was employed to model the heat loss term on the top surfaces of the substrate and melt pool regions [14].

**Finite element modeling of the functionally graded regions when additively manufactured by laser powder bed fusion and directed energy deposition**

The FactSage 8.1 program was used to analyze (1) the thermodynamic simulation based on the phase diagram, (2) the phase comparison, (3) the chemical decomposition, and (4) the thermal stability of each region in the FGS [16]. The tensile behaviors of the sequential LPBF-printed 48.1Al47.9Ti4.0V and DED-printed 48.1Al47.9Ti4.0V structures according to the horizontal (0°), diagonal (45°), and vertical (90°) BDs before HT were analyzed using a commercial finite element analysis (FEA) software package (Abaqus, Dassault Systèmes Simulia Corp., France), and the simulated stress−strain curves were compared with those obtained experimentally in the elastic regions [17]. A 3D CC was designed using CFD simulations in which a turbine blade incorporating the specific CC was additively manufactured using the hybrid LPBF and DED method, as presented in **Figures 8** and **9** [18, 19]. A commercial thermal transfer analysis software package (ANSYS mechanical 2019 R3, ANSYS, Inc., USA) was used to evaluate the cooling efficiency of the turbine blade with the specific type of conformal CC [18, 19].

**Topological optimization for developing a more advanced turbine blade system, complementary and synergistic incorporation of a lattice structure and cooling channel for additional mechanical stiffness and less thermal accumulation of each region on the functionally graded structure**

A 3D scanner (ZS−3040, Laser Design, USA) was used to replicate the original turbine blade used in the high-performance jet engine of an aircraft. The original part was fabricated using a combination of conventional manufacturing processes, such as casting, forging, and machining. To facilitate more accurate 3D scanning, an anti-glare powder was applied to the part's surface to reduce laser reflection [20]. The scanning data were captured as an ASC file containing point clouds [4]. This file was subsequently converted into an STL file by merging meshes, and then transformed into an STP file, which consists of faces and solids. Finite element modeling (FEM) was utilized to simulate the equivalent stress distributions in the original part, and TO was then applied to design the newly designed part that retained the stress-bearing regions of the original turbine blade while eliminating any unnecessary stress-free regions. In other words, TO minimizes the structural compliance (maximizes the deformation resistance), thus resulting in a reduced volume [7, 16, 21]. The technique employed herein is based on a solid isotropic material with penalization, which is dependent on mesh fidelity, and a penalized stiffness model is used to correlate volume and strength as follows:

$E\left( x \right) = {\rho(x)}^{p}E^{0}, \rho> 1$ and $\int_{\Omega} \rho\left( x \right)dΩ \leq V; 0 \leq\rho\left( x \right) \leq1, x \in\Omega$, (21)

where E is the elastic modulus of the element, ρ is the density of the element, p is the penalization factor, E^0^ is the original elastic modulus, Ω is the designed space domain that is eligible for optimization, and x is the individual element [7, 16, 21]. The total volume of the optimized domain must be equal to or less than the volume constraint V [7, 16, 21]. This equation iteratively modifies the relative density of each element in the FEM to determine the optimal spatial layout [21, 22]. If the element is not highly stressed, then the relative density decreases, because the element is not necessary for structural strength, whereas if the element is highly stressed, then its relative density increases to the initial value (full density) [23]. Meanwhile, an auxiliary lattice structure controlled by a hatching distance of 800 μm was deposited in the internal space of the topologically optimized turbine blade to maximize the mechanical stiffness of the spatial structure while minimizing the material usage [24]. This auxiliary structure was developed to maintain the overhang design that was created to increase the fuel efficiency of the aircraft used in this study. It is crucial to optimize the design of the conformal CC to improve the cooling efficiency of the FGS-based turbine blade and thus prevent thermal accumulation and extract thermal energy from the FGS [19, 25−27]. Consequently, a 3D CC was generated in the FGS by using CFD, and the turbine blade with a hierarchical CC was additively manufactured using the hybrid LPBF and DED method, along with the CNC system for subtractive manufacturing [28]. Quantitative data before and after TO were compared and, notably, the weight reduction was 8% without any mechanical stiffness change or thermal resistance drop.

**Microstructural comparison of each region in the functionally graded structure**

The mass transport, heat transfer, and fluid flow in the hybrid AM process for the 48.1Al47.9Ti4.0V bimodal structure built by LPBF and DED affect not only the track geometries after the laser scans, but also the sizes, morphologies, and distributions of the melt pools [14, 29]. In particular, for the combined AM process, the temperature gradient (G) and solidification rate (R_s_) are two critical factors that determine the sizes and morphologies of the melt pools over the solidified microstructures in both the LPBF and DED regions [14, 29]. Therefore, although the G/R_s_ values of the regions are similar, an increase in the G/R_s_ ratio leads to the formation of finer melt pools, whereas a decrease in this ratio results in the transformation of the solidified morphologies from planar to cellular, and from elongated to equiaxial [14, 29, 30]. Based on these two fundamental principles, in the numerical analyses of both the LPBF and DED processes for the 48.1Al47.9Ti4.0V bimodal structure, the fluid dynamics of the melt pools exhibited Newtonian motion with laminar flow [14, 31, 32]. As such, the enthalpy-porosity method was used to model the phase transition during the liquefaction and solidification processes [14, 31, 32]. Consequently, the heat-transfer and fluid-convection simulations of the 48.1Al47.9Ti4.0V bimodal structure that was additively manufactured using LPBF and DED under the optimized laser-control parameters (i.e., a constant laser power of 145 W and a scan speed of 16.7 mm min^−1^ for LPBF, and a constant laser power of 320 W and a scan speed of 1000 mm min^−1^ for DED) yielded the aforementioned steady-state melt pools devoid of microstructural defects and mechanical flaws, and ensured that a smooth surface was produced up to the end of the track, as shown in **Figure 2**. In other words, optimization of the LIr parameters of the combined AM process allowed for stable melt pool dynamics in the primary γ-based TiAl matrix phases with the secondary γ′-like Ti_3_Al precipitate phases. However, following the aforementioned principles, the Al_10_V and Al_45_V_7_ compounds at the dendrite and grain boundaries, were pushed out toward the IBs between the LPBF and DED regions [14, 33]. In addition, during LIr, the potential for the presence of segregates and precipitates at the edges of the melt pools increased along the heat-flow direction in the diffusive environment [34].

However, the results of another set of heat-transfer and fluid-convection simulations of pure Al when additively manufactured using the DED process with the lowest laser-control parameters (the lowest laser intensity of 5 W and highest scan speed of 50 mm s^−1^) are presented in **Figure S3**a. The 2D cross-sectional histograms of the single laser-scanned track of pure Al were plotted as observed from either the longitudinal or transverse directions, where each temperature gradient field was considered as a function of time for guiding the specific laser-control parameters [5, 35]. Due to extreme temperature variation depending on location within the melt pool, the melt-pool dynamics of pure Al became unsteady, and pores and cracks became abundant even at the start of the track, and this resulted in the evaporation and spattering of each constituent [14, 36]. The overheated environment induced by laser scanning led to the formation of a keyhole due to the excess energy imparted by the laser to the melt pools during the following two processes [14, 32]. First, the formation of pores proceeded via rapid collapse owing to vapor pressure on the surface of the laser-scanned track [37, 38]. Second, the inert Ar gas became trapped within the liquid metal, with the lagging shape flowing into the pores and cracks, thereby rapidly solidifying the melt pools. Otherwise, defects caused by the lack of fusion (**Figures S3**b and **S3**c) can occur, mainly due to improper infusion bonding between neighboring tracks on the powder bed during LPBF or the stream during DED. Based on these simulations, although the AM of pure Al was completed in practice, the process was extremely difficult because the evaporated Al formed a spatter [31, 39].

As depicted in **Figure S4**, the high TRs of the 48.1Al47.9Ti4.0V, 73.7Al24.2Ti2.1V, and 89.5Al10.0Ti0.5V structures were theoretically determined based on the Al−Ti, Al−V, and Ti−V phase diagrams, and were confirmed experimentally by the DSC−TGA analysis and tensile testing results at room and high temperatures. The hypothetical calculations (accounting for both the solid and liquid states) of the 48.1Al47.9Ti4.0V structure, regardless of whether LPBF or DED was applied, were equivalent to infinitely slow cooling or heating, in which the constituents of TiAl, Ti_3_Al, Al_10_V, and Al_45_V_7_ were also allowed to equilibrate at each temperature step. The most dominant TiAl compounds, each having a primitive tetragonal crystalline structure, were initially observed in the stable solid state at temperatures of up to 1389 °C, beyond which they began to liquify [40]. The secondary Ti_3_Al compounds, featuring a primitive hexagonal crystalline structure, started to transform into the primary TiAl compounds with the primitive tetragonal crystalline structure at the specific temperature of 374 °C, and melted simultaneously at 1389 °C [40, 41]. In comparison, the tertiary and quaternary Al_10_V and Al_45_V_7_ compounds were considerably more stable between 25 and 1511 °C without thermal decomposition, attributable to the presence of V, which has a considerably higher melting temperature of 1910 °C [42, 43]. For this reason, the liquid states of the V-based IMCs were not observed upon thermal decomposition of Al_10_V and Al_45_V_7_, or, at least, the quantity of V was maintained at temperatures of up to 1511 °C [42, 43]. As in the 48.1Al47.9Ti4.0V bimodal structure, several IMCs (TiAl_3_, TiAl_2_, Al_10_V, and Al_45_V_7_) of the 73.7Al24.2Ti2.1V and 89.5Al10.0Ti0.5V structures were present subsequently in the equilibrium states [44, 45]. However, starting at 660 °C, which is the melting temperature of Al, the pure Al and specific IMC phases (TiAl_3_, Al_10_V, and Al_45_V_7_) in the 89.5Al10.0Ti0.5V structure underwent distinct changes. Notably, the quantity of TiAl_3_ increased abruptly from 43.2 to 44.5 at.% and, thereafter, it melted completely at 1263 °C. Moreover, the V-based IMCs underwent thermal decomposition, as mentioned previously, whereas in case of the 73.7Al24.2Ti2.1V structure containing all the IMCs, the TiAl_3_ phases melted completely up to the temperature of 1339 °C. Meanwhile, the TiAl_2_ phases increased suddenly at 1309 °C and then decreased gradually up to 1372 °C [46, 47]. Taken together, these results indicated that after Al in TiAl_2_ was bound to another TiAl_3_, Al_10_V and Al_45_V_7_ moved toward each other in the temperature range of 1191−1372 °C, and both the compounds decomposed at higher temperatures, releasing liquid Al. Consequently, the TRs of the 48.1Al47.9Ti4.0V, 73.7Al24.2Ti2.1V, and 89.5Al10.0Ti0.5V structures were enhanced by the presence of Ti-containing IMCs with abnormally high melting temperatures relative to those of the conventional Al-based alloys, which were determined based on the results of thermal calculations relating to the specific regions in the FGS.

As deduced from the specific microstructures depicted in **Figures S5** and **S6**, although the heat generated by the LPBF process flowed directly along the 48.1Al47.9Ti4.0V structure, the flow of thermal energy was suppressed, with an accumulation of heat, owing to the presence of melt pools with low thermal conductivity; this resulted in a less distinct overlap of the IBs between the melt pools [48, 49]. Furthermore, only the IMC-comprised structure required a higher concentration of thermal energy compared to the conventional alloy-comprised structure, which potentially resulted in less bonding along the IBs between the matrix and precipitate phases, thereby attracting increasingly more thermal and residual stresses [50, 51]. In addition, thermal and residual stresses were present along the tertiary and quaternary compounds of Al_10_V and Al_45_V_7_, especially in the overlapping melt pools [17, 52]. This was primarily attributed to the presence of higher densities of voids, dislocations, and defects at the IBs between the melt pools, which reduced the strain and elongation [1, 53]. Nevertheless, when the secondary precipitate phases were tightly conjugated (entangled) with the primary matrix phases during LIr in the direction perpendicular to the melt pools, both in the finely and bluntly elongated dendrites and grains, the network environment that spread along the melt pools effectively helped to resist tensile deformation [52, 54, 55]. Similarly to the microstructures built using LPBF, the 48.1Al47.9Ti4.0V structure built using DED contained fan-shaped melt pools with finely elongated dendrites and grains at the centers and bluntly elongated dendrites and grains at the edges of the overlapping connections, which comprised the Ti_3_Al precipitate phases embedded in the TiAl matrix phases, as illustrated in **Figures S5**a−d [56, 57]. However, after HT (solid-solution treatment followed by aging), all of the melt pools had vanished from both the top and side planes of the microstructures (**Figure S5**e), leaving behind the more anisotropically unified lamellar γ-based matrix and α_2_-based precipitate phases, where the other Al_10_V and Al_45_V_7_ compounds did not undergo thermal decomposition [58, 59]. Consequently, considerably larger equiaxial dendrites and grains, along with more anisotropically aligned precipitate phases, were developed in the microstructures. Notably, the highly diffusive environment induced tighter bonding between the matrix and precipitate phases.

**Figure S6**e shows the characteristic 73.7Al24.2Ti2.1V microstructures consisting of (1) the γ-like matrix phases (TiAl_3_) with a body-centered tetragonal crystalline structure, (2) the γ′-like precipitate phases (TiAl_2_) with the identical crystalline structure, and (3) the segregates and precipitates percolated at the dendrite and grain boundaries [60, 61]. The inhomogeneous diffusion of Ti and V in the 73.7Al24.2Ti2.1V structure during fluid flow of the melt pools led to the formation of comparatively graded Ti- and V-rich regions, although their diameters ranged from a few tens of nanometers to several micrometers [62]. **Figure S6**e highlights the discernible differences in the distributions and concentrations of IMCs between the lighter TiAl_2_ and darker TiAl_3_ regions [63, 64]. **Figure S6**f illustrates the grain stabilization and precipitate dispersion roles of the γ′-like TiAl_3_ precipitate phases in the γ-based Al matrix phases of the 89.5Al10.0Ti0.5V structure [64, 65]. In the electron micrographs, pure Al phases were identified as smoother, darkened, and elongated areas within the melt pools. Moreover, comparatively brighter segregates and precipitates (emitting a greater number of secondary electrons), with irregular morphologies, were evident across the microstructures. The presence of IMCs in the dendrite and grain boundaries that are enriched with V is intuitive because V, which has a high melting temperature of 1910 °C, usually forms the most energetically stable IMCs with Al, and is classified as a grain-boundary stabilizer in the Al-based alloys [42, 43, 64]. The driving force for the segregation and precipitation caused by the V enrichment lowers the Gibbs free energy at the GBs [66]. However, the dendrites and grains of the 89.5Al10.0Ti0.5V structure, which are formed through rapid cooling during DED, constitute the heterogeneous constituents of the hierarchical microstructures, as demonstrated in **Figure S6**f [10].

The low-magnification microstructures of the Al-based FGS consisting of 48.1Al47.9Ti4.0V (by LPBF)/48.1Al47.9Ti4.0V (by DED)/73.7Al24.2Ti2.1V (by DED)/89.5Al10Ti0.5V (by DED) in the intended regions, which can be designed for a bimodal structure that requires the dual functionality provided by the different chemical compositions corresponding to the as-built positions, are depicted in **Figures S7**a and **S7**b. These layers were sequentially integrated in each step via a gradual transition from more Ti-dominant to more Al-dominant IMCs to achieve a compositional gradient, starting with the γ-based TiAl and γ′-like (α_2_-based) Ti_3_Al phases and passing through a series of discrete compositional mixtures (built by LPBF and subsequent DED) with the same phases in each layer, followed by other increments with considerably more Al-dominant layers consisting of IMCs with a composition of 73.7Al24.2Ti2.1V, and ending with the highest concentration of Al (89.5Al10Ti0.5V). However, after polishing and etching, each region of the FGS exhibited a microstructure similar to that of the conventional AM structure (**Figure S7**b), characterized by the elongated and equiaxial dendrites and grains and fan-shaped melt pools along the built layers, except for the distinctive IBs when the sequential LPBF (along 45°) and DED (along 90°) processes were used for the low-magnification microscopy analysis, primarily owing to the RS process after high-energy LIr, irrespective of the as-applied AM techniques. **Figure S7**c presents the Vickers hardness measurements across the FGS in the transformation from the 48.1Al47.9Ti4.0V structure built by LPBF to the 89.5Al10Ti0.5V structure built by DED. Although similar hardness values were provided for the bimodal 48.1Al47.9Ti4.0V structure obtained by consecutive LPBF and DED with a consistent (unaltered) chemical composition, a notable decrease can be observed along the specific regions where each of the powders with chemical compositions of 48.1Al47.9Ti4.0V, 73.7Al24.2Ti2.1V, and 89.5Al10Ti0.5V were deposited directly during LIr. In particular, the measured maximum Vickers hardness value of the FGS region with the composition of 48.1Al47.9Ti4.0V that was additively manufactured using LPBF was 611 HV_0.5_ owing to (1) the presence of brittle compounds (TiAl and Ti_3_Al) inside the dendrites and grains, along with the generation of large amounts of brittle V-based IMCs at the dendrite and grain boundaries, and (2) the formation of fewer pores and cracks when using LPBF compared to those obtained when using DED, despite the application of high-energy LIr during the two active diffusion processes. Similarly to the previous Vickers hardness measurements, the chemical compositions of the microstructures according to the as-built positions of the FGS are illustrated in **Figure S7**c. Although there were a few errors in the EDS analysis results, including those of the Ti-dominant IMCs in the lower build regions and the Al-dominant IMCs and alloys in the upper build regions, the molecular percentages of each constituent element (especially O) exhibited consistent increasing and decreasing trends. Accordingly, the as-built positions adequately matched the indentation locations created during the previous hardness measurements, thereby demonstrating significant correlations between the chemical compositions and hardness measurements.

**References**

1. Kim SH, Lee H, Yeon SM, Aranas C, Choi K, Yoon J, Yang SW, Lee H (2021) Selective compositional range exclusion via directed energy deposition to produce a defect-free Inconel 718/SS 316L functionally graded material. Addit Manuf 47:102288

2. Kim SH, Yeon SM, Kim JH, Park SJ, Lee JE, Park SH, Choi JP, Aranas C, Son Y (2019) Fine Microstructured In−Sn−Bi Solder for Adhesion on a Flexible PET Substrate: Its Effect on Superplasticity and Toughness. ACS Appl Mater Interfaces 11:17090−17099

3. Lee HB, Kim YW, Kim SH, Park SH, Choi JP, Aranas C (2018) A Modular Solder System with Hierarchical Morphology and Backward Compatibility. Small 14:1801349

4. Kim SH, Yeon SM, Lee JH, Kim YW, Lee H, Park J, Lee NK, Choi JP, Aranas C, Lee YJ, An S, Choi K, Son Y (2020) Additive manufacturing of a shift block via laser powder bed fusion: The simultaneous utilisation of optimised topology and a lattice structure. Virtual Phys Prototyp 15:460−480

5. Lin TC, Cao C, Sokoluk M, Jiang L, Wang X, Schoenung JM, Lavernia EJ, Li X (2019) Aluminum with dispersed nanoparticles by laser additive manufacturing. Nat Commun 10:4124

6. Aboulkhair NT, Simonelli M, Parry L, Ashcroft I, Tuck C, Hague R (2019) 3D printing of aluminium alloys: Additive manufacturing of aluminium alloys using selective laser melting. Prog Mater Sci 106:100578

7. Martin JH, Yahata BD, Hundley JM, Mayer JA, Schaedler TA, Pollock TM (2017) 3D printing of high-strength aluminium alloys. Nature 549:365−369

8. Park SJ, Heogh W, Yang J, Kang S, Jeong W, Lee H, Jang TS, Jung H Do, Jahazi M, Han SC, Kim HS, Lee MG, Bose S, Bandyopadhyay A, Jun MBG, Kim YW, Fu X, Advincula RC, Aranas C, Kim SH (2024) Meta-structure of amorphous-inspired 65.1Co28.2Cr5.3Mo lattices augmented by artificial intelligence. Adv Compos Hybrid Mater 7:224

9. Gong J, Wei K, Liu M, Song W, Li X, Zeng X (2022) Microstructure and mechanical properties of AlSi10Mg alloy built by laser powder bed fusion/direct energy deposition hybrid laser additive manufacturing. Addit Manuf 59:103160

10. Tiamiyu AA, Lucas T, Pang EL, Chen X, LeBeau JM, Schuh CA (2023) Heterogeneous microstructural evolution during hydrodynamic penetration of a high-velocity copper microparticle impacting copper. Mater Today 72:71−86

11. Laleh M, Sadeghi E, Revilla RI, Chao Q, Haghdadi N, Hughes AE, Xu W, De Graeve I, Qian M, Gibson I, Tan MY (2023) Heat treatment for metal additive manufacturing. Prog Mater Sci 133:101051

12. Khairallah SA, Martin AA, Lee JRI, Guss G, Calta NP, Hammons JA, Nielsen MH, Chaput K, Schwalbach E, Shah MN, Chapman MG, Willey TM, Rubenchik AM, Anderson AT, Morris Wang Y, Matthews MJ, King WE (2020) Controlling interdependent meso-nanosecond dynamics and defect generation in metal 3D printing. Science 368:660−665

13. Lee YS, Zhang W (2016) Modeling of heat transfer, fluid flow and solidification microstructure of nickel-base superalloy fabricated by laser powder bed fusion. Addit Manuf 12:178−188

14. Sun Z, Guo W, Li L (2020) Numerical modelling of heat transfer, mass transport and microstructure formation in a high deposition rate laser directed energy deposition process. Addit Manuf 33:101175

15. Wei HL, Mukherjee T, Zhang W, Zuback JS, Knapp GL, De A, DebRoy T (2021) Mechanistic models for additive manufacturing of metallic components. Prog Mater Sci 116:100703

16. Ghosh C, Aranas C, Jonas JJ (2016) Dynamic transformation of deformed austenite at temperatures above the Ae_3_. Prog Mater Sci 82:151−233

17. Pham MS, Liu C, Todd I, Lertthanasarn J (2019) Damage-tolerant architected materials inspired by crystal microstructure. Nature 565:305−311

18. Mostafaei A, Elliott AM, Barnes JE, Li F, Tan W, Cramer CL, Nandwana P, Chmielus M (2021) Binder jet 3D printing−process parameters, materials, properties, modeling, and challenges. Prog Mater Sci 119:100707

19. Heogh W, Yeon SM, Kang DS, Park S, Park S, Ryu K, Sun J, Ji L, Son Y, Choi K, Ha CW (2022) The design and additive manufacturing of an eco-friendly mold utilized for high productivity based on conformal cooling optimization. Mater Des 222:111088

20. Harding K (2008) Engineering precision. Nat Photonics 2:667−669

21. Benedetti M, du Plessis A, Ritchie RO, Dallago M, Razavi N, Berto F (2021) Architected cellular materials: A review on their mechanical properties towards fatigue-tolerant design and fabrication. Mater Sci Eng R 144:100606

22. Cui H, Yao D, Hensleigh R, Lu H, Calderon A, Xu Z, Davaria S, Wang Z, Mercier P, Tarazaga P, Zheng X (2022) Design and printing of proprioceptive three-dimensional architected robotic metamaterials. Science 376:1287−1293

23. Liu K, Sun R, Daraio C (2022) Growth rules for irregular architected materials with programmable properties. Science 377:975−981

24. Bayat M, Zinovieva O, Ferrari F, Ayas C, Langelaar M, Spangenberg J, Salajeghe R, Poulios K, Mohanty S, Sigmund O, Hattel J (2023) Holistic computational design within additive manufacturing through topology optimization combined with multiphysics multi-scale materials and process modelling. Prog Mater Sci 138:101129

25. Elhadad AA, Rosa-Sainz A, Cañete R, Peralta E, Begines B, Balbuena M, Alcudia A, Torres Y (2023) Applications and multidisciplinary perspective on 3D printing techniques: Recent developments and future trends. Mater Sci Eng R 156:100760

26. MacDonald E, Wicker R (2016) Multiprocess 3D printing for increasing component functionality. Science 353:aaf2093

27. Ravanji A, Lee A, Mohammadpour J, Cheng S (2023) Critical review on thermohydraulic performance enhancement in channel flows: A comparative study of pin fins. Renew Sustain Energy Rev 188:113793

28. Bandyopadhyay A, Traxel KD, Lang M, Juhasz M, Eliaz N, Bose S (2022) Alloy design via additive manufacturing: Advantages, challenges, applications and perspectives. Mater Today 52:207−224

29. Zhao C, Parab ND, Li X, Fezzaa K, Tan W, Rollett AD, Sun T (2020) Critical instability at moving keyhole tip generates porosity in laser melting. Science 370:849−852

30. Gu D, Shi X, Poprawe R, Bourell DL, Setchi R, Zhu J (2021) Material−structure−performance integrated laser-metal additive manufacturing. Science 372:eabg1487

31. Zekovic S, Dwivedi R, Kovacevic R (2007) Numerical simulation and experimental investigation of gas-powder flow from radially symmetrical nozzles in laser-based direct metal deposition. Int J Mach Tools Manuf 47:112−123

32. Wang L, Wang S, Zhang Y, Yan W (2023) Multi-phase flow simulation of powder streaming in laser-based directed energy deposition. Int J Heat Mass Transf 212:124240

33. Bayat M, Klingaa CG, Mohanty S, De Baere D, Thorborg J, Tiedje NS, Hattel JH (2020) Part-scale thermo-mechanical modelling of distortions in laser powder bed fusion−analysis of the sequential flash heating method with experimental validation. Addit Manuf 36:101508

34. Chen LY, Xu JQ, Choi H, Pozuelo M, Ma X, Bhowmick S, Yang JM, Mathaudhu S, Li XC (2015) Processing and properties of magnesium containing a dense uniform dispersion of nanoparticles. Nature 528:539−543

35. Bandyopadhyay A, Traxel KD, Bose S (2021) Nature-inspired materials and structures using 3D printing. Mater Sci Eng R 145:100609

36. Zhang K, Chen Y, Marussi S, Fan X, Fitzpatrick M, Bhagavath S, Majkut M, Lukic B, Jakata K, Rack A, Jones MA, Shinjo J, Panwisawas C, Leung CLA, Lee PD (2024) Pore evolution mechanisms during directed energy deposition additive manufacturing. Nat Commun 15:1715

37. Svetlizky D, Das M, Zheng B, Vyatskikh AL, Bose S, Bandyopadhyay A, Schoenung JM, Lavernia EJ, Eliaz N (2021) Directed energy deposition (DED) additive manufacturing: Physical characteristics, defects, challenges and applications. Mater Today 49:271−295

38. Lu X, Lin X, Chiumenti M, Cervera M, Hu Y, Ji X, Ma L, Yang H, Huang W (2019) Residual stress and distortion of rectangular and S-shaped Ti−6Al−4V parts by directed energy deposition: Modelling and experimental calibration. Addit Manuf 26:166−179

39. Arrizubieta JI, Lamikiz A, Cortina M, Ukar E, Alberdi A (2018) Hardness, grainsize and porosity formation prediction on the laser metal deposition of AISI 304 stainless steel. Int J Mach Tools Manuf 135:80−91

40. Musi M, Kardos S, Hatzenbichler L, Holec D, Stark A, Allen M, Güther V, Clemens H, Spoerk-Erdely P (2022) The effect of zirconium on the Ti−(42−46 at.%) Al system. Acta Mater 241:118414

41. Song L, Appel F, Liu W, Pyczak F, Zhang T (2023) {1$\bar{1}$01} tension twins and {1$\bar{1}$01}−{2$\bar{2}$01}/{2$\bar{2}$01}−{1$\bar{1}$01} double twins in the D0_19_ ordered hexagonal α_2_-Ti_3_Al phase. Acta Mater 260:119335

42. Shi C, Chen XG (2014) Effect of vanadium on hot deformation and microstructural evolution of 7150 aluminum alloy. Mater Sci Eng A 613:91−102

43. Wang F, Chiu YL, Eskin D, Du W, Shearing PR (2021) A grain refinement mechanism of cast commercial purity aluminium by vanadium. Mater Charact 181:111468

44. Sato H, Murase T, Fujii T, Onaka S, Watanabe Y, Kato M (2008) Formation of a wear-induced layer with nanocrystalline structure in Al−Al_3_Ti functionally graded material. Acta Mater 56:4549−4558

45. Hsu CJ, Chang CY, Kao PW, Ho NJ, Chang CP (2006) Al−Al_3_Ti nanocomposites produced in situ by friction stir processing. Acta Mater 54:5241−5249

46. Zhang LC, Palm M, Stein F, Sauthoff G (2001) Formation of lamellar microstructures in Al-rich TiAl alloys between 900 and 1100 °C. Intermetallics 9:229−238

47. Palm M, Zhang LC, Stein F, Sauthoff G (2002) Phases and phase equilibria in the Al-rich part of the Al−Ti system above 900 °C. Intermetallics 10:523−540

48. Wang YM, Voisin T, McKeown JT, Ye J, Calta NP, Li Z, Zeng Z, Zhang Y, Chen W, Roehling TT, Ott RT, Santala MK, Depond PJ, Matthews MJ, Hamza A V., Zhu T (2018) Additively manufactured hierarchical stainless steels with high strength and ductility. Nat Mater 17:63−71

49. Sanaei N, Fatemi A (2021) Defects in additive manufactured metals and their effect on fatigue performance: A state-of-the-art review. Prog Mater Sci 117:100724

50. Clarke AJ (2022) Unusual microstructures by 3D printing. Nat Mater 21:1223−1224

51. Saccone MA, Gallivan RA, Narita K, Yee DW, Greer JR (2022) Additive manufacturing of micro-architected metals via hydrogel infusion. Nature 612:685−690

52. Sing SL, Huang S, Goh GD, Goh GL, Tey CF, Tan JHK, Yeong WY (2021) Emerging metallic systems for additive manufacturing: In-situ alloying and multi-metal processing in laser powder bed fusion. Prog Mater Sci 119:100795

53. Zerbst U, Bruno G, Buffière JY, Wegener T, Niendorf T, Wu T, Zhang X, Kashaev N, Meneghetti G, Hrabe N, Madia M, Werner T, Hilgenberg K, Koukolíková M, Procházka R, Džugan J, Möller B, Beretta S, Evans A, Wagener R, Schnabel K (2021) Damage tolerant design of additively manufactured metallic components subjected to cyclic loading: State of the art and challenges. Prog Mater Sci 121:100786

54. du Plessis A, Razavi SMJ, Benedetti M, Murchio S, Leary M, Watson M, Bhate D, Berto F (2022) Properties and applications of additively manufactured metallic cellular materials: A review. Prog Mater Sci 125:100918

55. Shang X, Liu Z, Zhang J, Lyu T, Zou Y (2023) Tailoring the mechanical properties of 3D microstructures: A deep learning and genetic algorithm inverse optimization framework. Mater Today 70:71−81

56. Huang D, Tan Q, Zhou Y, Yin Y, Wang F, Wu T, Yang X, Fan Z, Liu Y, Zhang J, Huang H, Yan M, Zhang MX (2021) The significant impact of grain refiner on γ-TiAl intermetallic fabricated by laser-based additive manufacturing. Addit Manuf 46:102172

57. Zhang X, Li C, Wu M, Ye Z, Wang Q, Gu J (2022) Atypical pathways for lamellar and twinning transformations in rapidly solidified TiAl alloy. Acta Mater 227:117718

58. Nó ML, Klein T, Clemens H, San Juan JM (2023) High-temperature microstructure evolution of an advanced intermetallic nano-lamellar γ-TiAl-based alloy and associated diffusion processes. Acta Mater 261:119380

59. Song L, Appel F, Wang L, Oehring M, Hu X, Stark A, He J, Lorenz U, Zhang T, Lin J, Pyczak F (2020) New insights into high-temperature deformation and phase transformation mechanisms of lamellar structures in high Nb-containing TiAl alloys. Acta Mater 186:575−586

60. Chen G, Shu X, Liu J, Zhang B, Feng J (2020) A new coating method with potential for additive manufacturing: Premelting electron beam-assisted freeform fabrication. Addit Manuf 33:101118

61. Jiang F, Tang L, Ye H, Yang Z (2023) The role of TiH_2_ on microstructure and mechanical properties of Al−Zn−Mg−Cu alloy fabricated by laser powder bed fusion. Mater Sci Eng A 869:144819

62. Qu Z, Zhang Z, Liu R, Xu L, Zhang Y, Li X, Zhao Z, Duan Q, Wang S, Li S, Ma Y, Shao X, Yang R, Eckert J, Ritchie RO, Zhang Z (2024) High fatigue resistance in a titanium alloy via near-void-free 3D printing. Nature 626:999−1004

63. Ohnuma I, Fujita Y, Mitsui H, Ishikawa K, Kainuma R, Ishida K (2000) Phase equilibria in the Ti−Al binary system. Acta Mater 48:3113−3123

64. Galano M, Audebert F (2022) Novel Al based nanoquasicrystalline alloys. Prog Mater Sci 123:100831

65. Sequeira PD, Watanabe Y, Fukui Y (2005) Backward extrusion of Al−Al_3_Ti functionally graded material: Volume fraction gradient and anisotropic orientation of Al_3_Ti platelets. Scr Mater 53:687−692

66. George EP, Raabe D, Ritchie RO (2019) High-entropy alloys. Nat Rev Mater 4:515−534

67. Lin C, Wu W, Han Y, Liu J, Zhang M, Wang Q, Li X (2023) Orderly nucleation and competitive growth behaviors of Ti−Al intermetallic compounds in Ti/TiAl_3_ diffusion couple under high temperature. J Alloys Compd 939:168815

68. Ren J, Zhang Y, Zhao D, Chen Y, Guan S, Liu Y, Liu L, Peng S, Kong F, Poplawsky JD, Gao G, Voisin T, An K, Wang YM, Xie KY, Zhu T, Chen W (2022) Strong yet ductile nanolamellar high-entropy alloys by additive manufacturing. Nature 608:62−68

69. Akbarpour MR, Mirabad HM, Hemmati A, Kim HS (2022) Processing and microstructure of Ti−Cu binary alloys: A comprehensive review. Prog Mater Sci 127:100933

70. Zhang D, Qiu D, Gibson MA, Zheng Y, Fraser HL, StJohn DH, Easton MA (2019) Additive manufacturing of ultrafine-grained high-strength titanium alloys. Nature 576:91−95

71. Plotkowski A, Rios O, Sridharan N, Sims Z, Unocic K, Ott RT, Dehoff RR, Babu SS (2017) Evaluation of an Al−Ce alloy for laser additive manufacturing. Acta Mater 126:507−519

72. Zhao J, Wang X, Miao D, Xue F, Zhou J (2023) Grain refinement of Fe_3_Al-based iron aluminide fabricated using wire-arc additive manufacturing. Mater Lett 349:134795

73. Shen C, Pan Z, Ma Y, Cuiuri D, Li H (2015) Fabrication of iron-rich Fe−Al intermetallics using the wire-arc additive manufacturing process. Addit Manuf 7:20−26

74. Khomutov M, Potapkin P, Cheverikin V, Petrovskiy P, Travyanov A, Logachev I, Sova A, Smurov I (2020) Effect of hot isostatic pressing on structure and properties of intermetallic NiAl−Cr−Mo alloy produced by selective laser melting. Intermetallics 120: 106766

75. Meng Y, Li J, Zhang S, Gao M, Gong M, Chen H (2023) Wire arc additive manufacturing of Ni−Al intermetallic compounds through synchronous wire-powder feeding. J Alloys Compd 943:169152

76. Meng Y, Li J, Gao M, Zeng X (2021) Microstructure characteristics of wire arc additive manufactured Ni−Al intermetallic compounds. J Manuf Process 68:932−939

77. Han J, Chen X, Zhang G, Liu B, Cai Y, Chen M, Jiang H, Tian Y (2023) Ni_50.8_Ti_49.2_ alloy prepared by double-wire + arc additive manufacturing with a substrate heating temperature of 600 °C. J Manuf Process 104:76−86

78. Mitra I, Bose S, Dernell WS, Dasgupta N, Eckstrand C, Herrick J, Yaszemski MJ, Goodman SB, Bandyopadhyay A (2021) 3D printing in alloy design to improve biocompatibility in metallic implants. Mater Today 45:20−34

**Table S1.** A comparison of the LIr parameters and AM variables at each transition range of the FGS with a compositional ratio of 48.1Al47.9Ti4.0V/73.7Al24.2Ti2.1V/89.5Al10.0Ti0.5V having a nominal chemical composition.

| **Distance**  **(mm)** | **Ti amount**  **(at.%)** | **Al amount**  **(at.%)** | **V amount**  **(at.%)** | **O amount**  **(at.%)** | **Laser intensity**  **(W)** | **Laser speed**  **(mm min^−1^)** | **Flow rate**  **(L min^−1^)** |
| --- | --- | --- | --- | --- | --- | --- | --- |
| −11 | 47.23 | 47.59 | 4.01 | 0.42 | 145 | 16.7 | − |
| −9 | 47.27 | 47.94 | 4.02 | 0.39 | 145 | 16.7 | − |
| −7 | 47.88 | 48.11 | 3.98 | 0.43 | 145 | 16.7 | − |
| −5 | 47.92 | 48.13 | 3.98 | 0.45 | 145 | 16.7 | − |
| −3 | 47.25 | 48.05 | 4.05 | 0.45 | 145 | 16.7 | − |
| −1 | 47.05 | 47.37 | 4.03 | 0.58 | 145 | 16.7 | − |
| 1 | 47.19 | 47.31 | 3.97 | 0.61 | 320 | 1000.0 | 45.5 |
| 3 | 45.47 | 49.12 | 3.85 | 0.69 | 305 | 1052.5 | 42.0 |
| 5 | 44.34 | 51.06 | 3.72 | 0.43 | 290 | 1105.0 | 38.5 |
| 7 | 42.98 | 53.89 | 3.60 | 0.68 | 275 | 1157.5 | 35.0 |
| 9 | 40.01 | 55.01 | 3.44 | 0.61 | 260 | 1210.0 | 31.5 |
| 11 | 38.95 | 57.04 | 3.29 | 0.53 | 245 | 1262.5 | 28.0 |
| 13 | 36.33 | 59.01 | 3.17 | 0.52 | 230 | 1315.0 | 24.5 |
| 15 | 34.98 | 61.02 | 3.03 | 0.62 | 215 | 1367.5 | 21.0 |
| 17 | 32.01 | 63.08 | 2.87 | 0.77 | 200 | 1420.0 | 17.5 |
| 19 | 30.09 | 65.50 | 2.74 | 0.73 | 185 | 1472.5 | 14.0 |
| 21 | 28.18 | 67.19 | 2.62 | 0.83 | 170 | 1525.0 | 10.5 |
| 23 | 27.01 | 69.37 | 2.43 | 0.73 | 155 | 1577.5 | 7.0 |
| 25 | 25.13 | 71.04 | 2.24 | 1.04 | 140 | 1630.0 | 3.5 |
| 27 | 23.98 | 73.25 | 2.12 | 0.76 | 125 | 1682.5 | 45.5 |
| 29 | 21.95 | 75.54 | 2.03 | 0.76 | 115 | 1735.0 | 39.0 |
| 31 | 19.17 | 77.05 | 1.73 | 0.83 | 105 | 1787.5 | 32.5 |
| 33 | 17.05 | 80.38 | 1.45 | 0.77 | 95 | 1840.0 | 26.0 |
| 35 | 15.25 | 82.84 | 1.19 | 0.97 | 85 | 1892.5 | 19.5 |
| 37 | 13.14 | 84.76 | 0.91 | 0.72 | 75 | 1945.0 | 13.0 |
| 39 | 11.06 | 86.40 | 0.68 | 0.80 | 65 | 1997.5 | 6.5 |
| 41 | 9.98 | 88.89 | 0.45 | 0.93 | 55 | 2050.0 | 45.5 |

**Table S2.** A reference comparison of the physical and chemical properties of the additively manufactured structures with those of the conventional structures fabricated by casting, welding, spraying, etc., evaluated and classified in terms of lightweight alloy design.

| **AM**  **process** | **Alloy**  **system** | **Density**  **(g cm^−3^)** | **Specific applications** | **Manufacturing advantages** | **Mechanical**  **properties** | **Thermal**  **resistance** | **Key**  **points** | **Ref.**  **(No.)** |
| --- | --- | --- | --- | --- | --- | --- | --- | --- |
| O | Al−Ti | 2.9−3.7 | Turbine blade | The design feedback loop can be very agile in the majority of laser-based AM techniques and methods due to their facile instrument setups, even in the middle of the production line | FGS with high UTS (0.5−1.7 GPa) | Its TR is intrinsically high (450−900 °C) owing to the compositions of the constituent IMCs | FGS | Our study |
| X | Al−Ti (the formation of TiAl_3_) | 3.2 | The most promising candidate material for use in high-temperature structural applications in the aerospace and automobile industries | Vacuum hot press sintering of Al and Ti foils | The Ti_3_Al phase possesses the highest hardness of 11.1 GPa, the hardness and elastic modulus of the TiAl_3_ phase are 9.8 GPa and 200 GPa, respectively, and those of the Ti_2_Al_5_ phase are 7.3 GPa and 181 GPa | Volume diffusion, grain diffusion, and simultaneous chemical reaction and volume diffusion occurred at 950, 1050, and 1150 °C, respectively | Paucity of published research and difficult precursor fabrication | [67] |
| O (DED) | Ti−44Al−4Nb−1Mo−1Cr (micro-sized) and LaB_6_ nanoparticles (100 nm) | 3.7 | Particularly when used in the aerospace industry | The mass ratio of 99.5:0.5 was found to be optimal for minimizing the amount of LaB_6_ added, and mechanical agitation was performed for 2 h to ensure mixing homogeneity to provide the highest mechanical strength | The YS, UTS, and strain of the as-printed TiAl alloy were 842.9 MPa, 1806.9 MPa, and 11.5%, respectively; however, the corresponding values of the as-printed TiAl−LaB_6_ composite were 1089.3 MPa, 2031.6 MPa, and 18.6% | The authors found that the γ-based TiAl IMCs possessed excellent oxidation resistance even at temperatures exceeding 800 °C | The rare-earth element La stemming from the decomposition of LaB_6_ is effectively used as an oxygen scavenger in the γ-based TiAl alloy because of their stronger affinity to O than to Ti and Al, as evidenced by the formation of La_2_O_3_ | [56] |
| O (LPBF) | AlCoCrFeNi2.1 eutectic high-entropy alloy | 7.0−9.0 | No applications | A high cooling rate under high-energy LIr can increase strength and reduce elongation in the high-entropy alloy system before HT | The tensile YS of the as-printed sample is 1333 MPa, which is substantially higher than that of the as-cast sample (510 MPa) | The TS and strain were 1907 MPa and 7.4%, respectively, after annealing at 600 °C for 5 h and 1286 MPa and 23.8% after annealing at 800 °C for 1 h | Possessing both an intrinsically high strength and high density (low specific strength) | [68] |
| X and O (various manufacturing techniques and methods) | Ti−Cu | 4.5−9.0 | Biomedical devices and implants | The mechanical properties provided by most of the materials and structures developed using the laser-based AM techniques and methods are potentially comparable to those provided by the traditional cast-based approaches after HT | The TSs and strains of Ti−3.5Cu, Ti−6.5Cu, and Ti−8.5Cu alloys are 867 MPa and 14.9%, 964 MPa and 5.5%, and 1023 MPa and 2.1%, respectively | Ti_2_Cu has a melting temperature of 1005 °C | Antibacterial activity and biocompatibility are achieved by developing various Ti_x_Cu_y_ IMCs | [69, 70] |
| X and O (LPBF and DED) | Al−Ce | 2.7−6.8 | Al−Ce alloys show promise for use in high-temperature, weight-critical applications such as automotive engines | Limited due to scarce alloying or rare-earth elements that are highly expensive to procure in powder, wire, or filament forms, along with a lack of research on these highly exotic alloys | The as-cast Al−12Ce binary alloy had a YS of 57 MPa, UTS of 161 MPa, and elongation of 13.5%; however, T6 HT tended to decrease the UTS and YS by approximately 20%, but it nearly doubled ductility to 26.5% | Al−Ce alloys may be stable even when exposed to temperatures exceeding 300 °C, and possibly at higher temperatures, and given their excellent thermal stability and good castability, they are good candidates for AM | The thermodynamically modeled eutectic spacing and microstructures were consistent with those obtained in the experiments involving remelting under the as-cast and AM-representative conditions | [71] |
| O (wire-arc AM) | Al−Fe | 2.7−7.9 | − | The high cooling rate can lead to a few challenges and requirements when using novel strategies for the as-printed structure and controlling its microstructures | The UTS, YS, and elongation of AlFe_3_ are 950 MPa, 850 MPa, and 3.3%, respectively | The high TR was attributed to the presence of iron, which has a high melting temperature of 1538 °C | An abundance of brittle precipitates can result in the formation of high-hardness microstructures and induce cracking | [72, 73] |
| O (wire-arc AM and LPBF) | Al−Ni (Ni_3_Al or NiAl) | 5.9 (NiAl) | The material has broad application prospects in the aerospace, furnace equipment, and automotive industries | When using powder materials, the high deposition efficiency and low manufacturing cost of wire-arc AM can address the easy oxidation, high cost, and low efficiency of laser AM | Compression testing results of the selective laser melting samples after hot isostatic pressing indicated that their YS decreased from 750 to 260 MPa when the temperature increased from 700 to 1100 °C | The AM structures exhibit a lower density (5.9 g cm^−3^), higher melting point (1638 ℃), better thermal conductivity (78 W m^−1^ K^−1^), and excellent oxidation/corrosion resistance compared to those of typical alloys | Needle-like NiAl and lamellar Ni_3_Al precipitates were observed on the lath-shaped NiAl | [74−76] |
| O (double-wire-arc AM) | Ni−Ti | 6.7 | Various industries, including the biomedical, aerospace, and construction industries | Double wire-arc AM allows for the fabrication of alloys by using different raw materials, thereby broadening the range of possible materials | The hardness range was 200−550 HV_0.2_ | High TR owing to the presence of Ni, which has a high melting temperature of 1455 °C | The results revealed that Ni_4_Ti_3_ was precipitated mainly in spherical and needle-like forms, which contributed to an improved component strength via coherent strain, strengthening, and lattice distortion | [77] |
| O (DED) | Ti−Ta | > 4.5 | Biomedical applications | By using powder blending and multi-alloy hopper via DED, good agreement can be achieved between the predicted and as-printed phase compositions | A significant difference was observed between 10Ta−P and 25Ta−P owing to the addition of Ta to Ti | Is not necessary for biomedical applications | The addition of as low as 10 wt.% Ta induced a strong biological response comparable to that achieved by the addition of 100 wt.% Ta | [78] |

**Table S3.** The physical and chemical properties of the FGS in each transition range in the variables for a compositional ratio of 48.1Al47.9Ti4.0V/73.7Al24.2Ti2.1V/89.5Al10.0Ti0.5V with a nominal chemical composition.

| **Chemical Comp.**  **(at.%)** | **O amount**  **(at.%)** | **C amount**  **(at.%)** | **Vickers H.**  **(HV_0.5_)** | **Exp. D.**  **(g cm^−3^)** | **Melting T.**  **(°C)** | **Tensile S.**  **(MPa)** | **Elastic M.**  **(GPa)** |
| --- | --- | --- | --- | --- | --- | --- | --- |
| 47.59−47.23−4.01 | 0.42 | 0.002 | 601 | 3.71 | 1460 | 1679 | 231.9 |
| 47.94−47.27−4.02 | 0.39 | 0.002 | 603 | 3.71 | 1460 | 1679 | 231.9 |
| 48.11−47.88−3.98 | 0.43 | 0.001 | 608 | 3.71 | 1460 | 1679 | 231.9 |
| 48.13−47.92−3.98 | 0.45 | 0.001 | 611 | 3.71 | 1460 | 1679 | 231.9 |
| 48.05−47.25−4.05 | 0.45 | 0.003 | 604 | 3.71 | 1460 | 1679 | 231.9 |
| 47.37−47.05−4.03 | 0.58 | 0.002 | 590 | 3.71 | 1460 | 1679 | 231.9 |
| 47.31−47.19−3.97 | 0.61 | 0.001 | 591 | 3.71 | 1460 | 1513 | 220.8 |
| 49.12−45.47−3.85 | 0.69 | 0.001 | 582 | 3.67 | − | − | − |
| 51.06−44.34−3.72 | 0.43 | 0.002 | 573 | 3.63 | − | − | − |
| 53.89−42.98−3.60 | 0.68 | 0.002 | 565 | 3.60 | − | − | − |
| 55.01−40.01−3.44 | 0.61 | 0.001 | 567 | 3.56 | − | − | − |
| 57.04−38.95−3.29 | 0.53 | 0.001 | 549 | 3.52 | − | − | − |
| 59.01−36.33−3.17 | 0.52 | 0.001 | 544 | 3.48 | − | − | − |
| 61.02−34.98−3.03 | 0.62 | 0.001 | 531 | 3.45 | − | − | − |
| 63.08−32.01−2.87 | 0.77 | 0.001 | 532 | 3.41 | − | − | − |
| 65.50−30.09−2.74 | 0.73 | 0.001 | 524 | 3.37 | − | − | − |
| 67.19−28.18−2.62 | 0.83 | 0.001 | 525 | 3.33 | − | − | − |
| 69.37−27.01−2.43 | 0.73 | 0.001 | 516 | 3.30 | − | − | − |
| 71.04−25.13−2.24 | 1.04 | 0.001 | 507 | 3.26 | − | − | − |
| 73.25−23.98−2.12 | 0.76 | 0.001 | 493 | 3.22 | 1340 | 1331 | 187.6 |
| 75.54−21.95−2.03 | 0.76 | 0.001 | 463 | 3.17 | − | − | − |
| 77.05−19.17−1.73 | 0.83 | 0.001 | 437 | 3.13 | − | − | − |
| 80.38−17.05−1.45 | 0.77 | 0.001 | 321 | 3.08 | − | − | − |
| 82.84−15.25−1.19 | 0.97 | 0.001 | 207 | 3.04 | − | − | − |
| 84.76−13.14−0.91 | 0.72 | 0.001 | 131 | 2.99 | − | − | − |
| 86.40−11.06−0.68 | 0.8 | 0.001 | 136 | 2.95 | − | − | − |
| 88.89−9.98−0.45 | 0.93 | 0.001 | 129 | 2.90 | 664 | 609 | 104.5 |
| Pure Al | 0.97 | 0.001 | 31 | 2.70 | 660 | 90 | 62.5 |

**Table S4.** The coefficients of turbulence used for the default setting values during the ANSYS analysis. The coefficients applied in the specific turbulence model, and the FEA conditions used in the specific turbulence model.

| **Models** |  |
| --- | --- |
| Solver | Pressure-based |
| Velocity formulation | Absolute |
| Time | Steady |
| Viscous model | Realizable k−ε model (2 eqn.) |
|  |  |
| **Boundary conditions** |  |
| Wall shear condition | No slip |
|  |  |
| **Solution methods** |  |
| Scheme | Coupled |
| Flux type | Distance based |
| Gradient | Least squares cell based |
| Pressure | Second order |
| Momentum | Second order upwind |
| Turbulent kinetic energy | First order upwind |
| Specific dissipation rate | First order upwind |
|  |  |
| **Calculations** |  |
| Length scale method | Conservative |
| Number of iterations | 500 |
| Reporting interval | 1 |

**Table S5.** A comparison of the parameters with regard to the coolant dynamics in cases 1−4. A comparison of the variable parameters d_1−4_ and z_1−4_ in cases 1−4, respectively, in terms of the Re_N_ relative to the flow uniformity of the coolant air on the basis of the CFD analysis results of these cases.

| **Case** | **d (mm)** | **z (mm)** | **Avg. Re_N_ (unitless)** | **Std. dev. (unitless)** |
| --- | --- | --- | --- | --- |
| **Case 1** | − | − | 22.4 | 17.8 |
| **Case 2** | 42 | 6 | 85.9 | 14.0 |
| **Case 3** | 6 | 6 | 135.6 | 10.3 |
| **Case 4** | 3 | 3 | 41.4 | 16.5 |


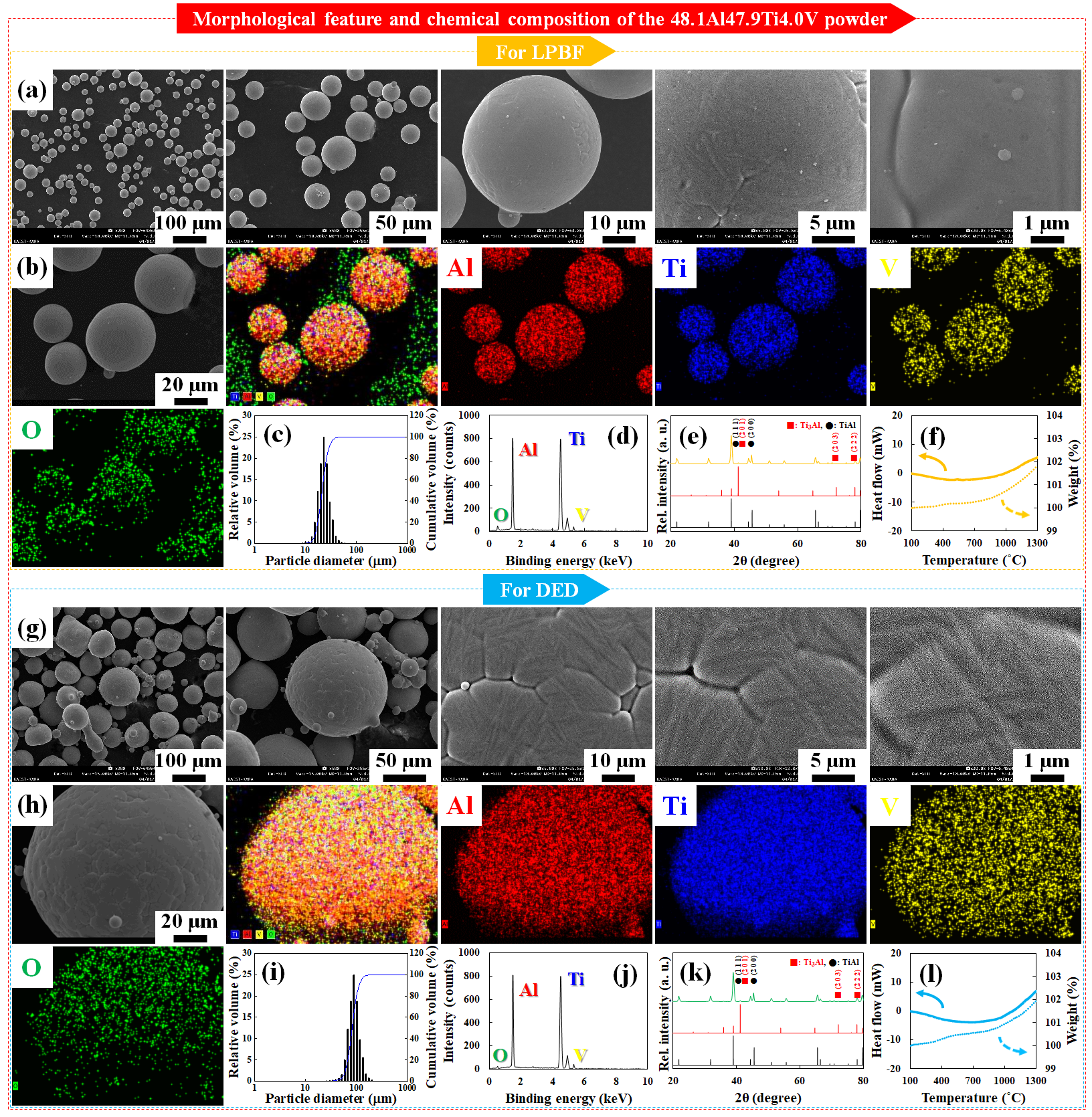


**Figure S1.** (a–f) Characterization of the 48.1Al47.9Ti4.0V powder built by LPBF: (a) SEM images at various magnifications, (b) an SEM image and corresponding elemental mapping images, (c) the particle size distribution, (d) the EDS spectrum, (e) the XRD pattern, and (f) the DSC scan. The powder consisted of spherical particles with a median diameter of 36 μm, which were mainly composed of Al, Ti, V, and O. (g–l) Characterization of the 48.1Al47.9Ti4.0V powder built by DED: (g) SEM images at various magnifications, (h) an SEM image and corresponding elemental mapping images, (i) the particle size distribution, (j) the EDS spectrum, (k) the XRD pattern, and (l) the DSC scan. Similarly, the powder had spherical particles, consisting of Al, Ti, V, and O, but with a much bigger median diameter of 94 μm. Owing to the high pressure (30 bar) of gas atomization towards the 48.1Al47.9Ti4.0V liquid droplet, spherization of the powder particles used in both the LPBF and DED processes was induced as the most energetically stable state. Furthermore, the surfaces of both powder particles were fully infilled and packed inside the cores without any pores and cracks, and they solidified rapidly at the high cooling rates of 10^3^−10^7^ K s^−1^ and then became more densified during HT at a temperature of 480 °C. Despite the barely detectable amounts and small sizes of the TiAl and Ti_3_Al phases, the XRD and DSC analyses demonstrate that these were retained in both powder particles, and there were no unexpected IMCs and V-based compounds.


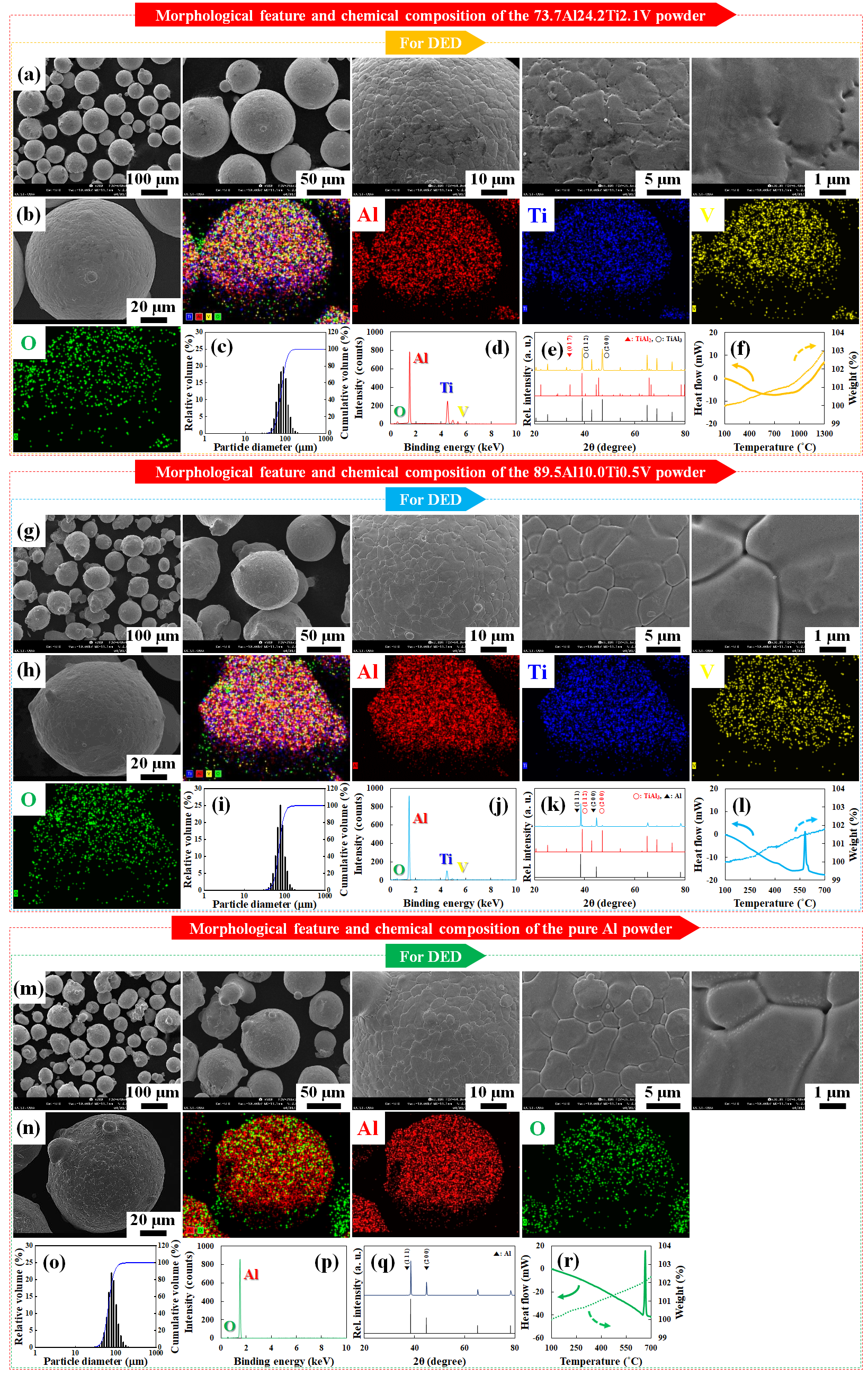


**Figure S2.** (a–f) Characterization of the 73.7Al24.2Ti2.1V powder built by DED: (a) SEM images at various magnifications, (b) an SEM image and corresponding elemental mapping images, (c) the particle size distribution, (d) the EDS spectrum, (e) the XRD pattern, and (f) the DSC scan. The powder consisted of spherical particles with a median diameter of 93 μm, which were mainly composed of Al, Ti, V, and O. (g–l) Characterization of the 89.5Al10.0Ti0.5V powder built by DED: (g) SEM images at various magnifications, (h) an SEM image and corresponding elemental mapping images, (i) the particle size distribution, (j) the EDS spectrum, (k) the XRD pattern, and (l) the DSC scan. This powder consisted of spherical particles, which were composed of Al, Ti, V, and O, with a similar median diameter of 91 μm. (m–q) Characterization of the pure Al powder built by DED: (m) SEM images at various magnifications, (n) an SEM image and corresponding elemental mapping images, (o) the particle size distribution, (p) the EDS spectrum, (q) the XRD pattern, and (r) the DSC scan. The last powder had spherical particles, consisting of Al and O, with a slightly smaller median diameter of 89 μm. The powder particles exhibited smooth surfaces because nucleate formation and phase growth were impossible during the RS process, even though they had been heat treated to further relieve their generated thermal and residual stresses at 480, 440, and 220 °C for 30 min each, and finally, slowly cooled to 25 °C in a furnace.

**Figure S3.** (a) A 3D view of the single laser track on the pure Al powder stream with the temperature gradient field showed many microstructural defects and mechanical flaws, even when the minimum laser power of 5 W and maximum scan speed of 50 mm s^−1^ were applied. (b, c) 2D cross-sectional histograms of the single laser track on the pure Al phases from either the horizontal or vertical directions with each temperature gradient field as a function of time. Thus, this AM process was unsuccessful because of the formation of abundant microstructural defects and mechanical flaws, irrespective of the applied laser parameters.

**Figure S4.** The Al−Ti (a), Al−V (b), and Ti−V (c) phase diagrams validated theoretically the high TRs of the 48.1Al47.9Ti4.0V, 73.7Al24.2Ti2.1V, and 89.5Al10.0Ti0.5V structures. (d) The equilibrium phase fractions of the 48.1Al47.9Ti4.0V structure confirmed the presence of IMCs with high TRs, including various solid and liquid phases. (e) The equilibrium phase fractions of the 73.7Al24.2Ti2.1V structure were calculated in the presence of γ-like TiAl_3_ and γ′-like Ti_2_Al in addition to the V-based compounds (Al_10_V and Al_45_V_7_) while including various solid and liquid phases. (f) The equilibrium phase fractions of the 89.5Al10.0Ti0.5V structure were calculated in the presence of γ-based Al and γ′-like TiAl_3_ in addition to the identical chemical compositions of Al_10_V and Al_45_V_7_, while including various solid and liquid phases simultaneously. Note that (g), (h), and (i) are the expanded regions of (d), (e), and (f), respectively, in the compositional ratios of Al, Ti, and V. However, pure Al is energetically unstable at such high temperatures. For this reason, it melted at temperatures of up to 660 °C and started to evaporate at 2470 °C. The equilibrium phase fractions in each region of the FGS confirmed both the formation of IMCs as by-products owing to the active diffusion of Ti and V, and the high TRs of all these IMCs. However, only the 89.5Al10.0Ti0.5V structure had a low TR owing to the low amount of TiAl_3_. Nevertheless, because strong covalent bonds were formed between the constituent elements of the other IMCs, those phases were relatively stable and did not decompose until the IMCs disintegrated and separated into each constituent element. Thus, all the regions of the FGS, except for that composed of pure Al, retained their high TRs despite their Al-based chemical compositions.

**Figure S5.** SEM images showing the side-plane microstructures at the IBs of the bimodal structures when additively manufactured by the sequential LPBF and DED processes with the BDs of 0°/90°, 45°/90°, and 90°/90°, respectively, before and after HT. The specific regions in the FGS were divided by intersectional crossing with the 45° direction along the centers of the melt pools when additively manufactured by LPBF, which generated fewer microstructural defects and mechanical flaws. Subsequently, when the DED process was used to build vertically along the 90° direction, a higher contact area was generated in the FGS compared to that obtained when using LPBF to build along the 0° and 90° directions in more contact with the edges of the melt pools. However, after HT (solid-solution treatment followed by aging), all of the melt pools had disappeared from both the top and side planes of the microstructures, leaving the more unified α_2_-based Ti_3_Al precipitate phases without any thermal decomposition of the V-based (Al_10_V and Al_45_V_7_) compounds throughout the γ-based TiAl matrix phases. Subsequently, although the continuous and high-temperature environment provided tighter bonding between the matrix and precipitate phases, more aligned precipitate phases were developed within larger equiaxial dendrites and grains throughout the microstructures.

**Figure S6.** (c) SEM images showing the microstructures of the additively manufactured 48.1Al47.9Ti4.0V structure built by LPBF. The side-plane view clearly demonstrates the hemi-ellipsoidal melt pools (red dashed lines) spreading outward opposite to the BD. (d) SEM images showing the microstructures of the additively manufactured 48.1Al47.9Ti4.0V structure built by DED. (e) SEM images showing the microstructures in the side plane of the additively manufactured 73.7Al24.2Ti2.1V structure built by DED. (f) SEM images showing the microstructures in the side plane of the additively manufactured 89.5Al10.0Ti0.5V structure built using DED. The TiAl_3_, Al_10_V, and Al_45_V_7_ precipitates were pushed outward to the GBs and melt pools of the pure Al matrix phases in the diffusive environment during LIr, and solidified rapidly at the edges of the melt pools. The SEM images of the microstructures in the side planes of (g) the 48.1Al47.9Ti4.0V structure built using LPBF, the 48.1Al47.9Ti4.0V structure built using DED, (h) the 73.7Al24.2Ti2.1V structure built using DED, and (i) the 89.5Al10.0Ti0.5V structure built using DED after subsequent HT (solid-solution treatment at 1100, 1000, and 450 °C for 30 min each, followed by rapid cooling to 25 °C within 10 min by means of gas quenching). These cooled structures were then aged at 480, 440, and 220 °C for 30 min each, and finally, slowly cooled to 25 °C in a furnace to (1) alleviate the thermal and residual stresses generated during LIr and those accumulated because of gas quenching due to the solid-solution treatment, (2) reduce the numbers of microstructural defects and mechanical flaws, and (3) transform their heterogeneous phases into more homogeneous microstructures despite the presence of abundant IMCs.

**Figure S7.** (a, b) Panoramic SEM images showing the Al-based FGS before HT. (c) Before HT, the Vickers hardness values and the quantity of V-based compounds measured along the functionally graded microstructures decreased simultaneously up to the end of the as-built FGS. By contrast, a few of their LPBF- and DED-printed regions with the chemical composition of 48.1Al47.9Ti4.0V remained virtually intact compared to the other regions with the chemical compositions of 73.7Al24.2Ti2.1V and 89.5Al10.0Ti0.5V. The Vickers hardness values decreased from the 48.1Al47.9Ti4.0V structure to the 89.5Al10.0Ti0.5V structure because the amounts of Al_10_V and Al_45_V_7_ compounds with high hardness values that were formed in the FGS decreased gradually, as did the amounts of Ti-containing IMCs, regardless of the use of LPBF or DED. (d) The density of each region in the FGS decreased gradually as the quantity of Al-based compounds increased. (e−f) The DSC−TGA analysis revealed that the structures containing the aforementioned IMCs possessed high TRs, even at temperatures of up to 450−900 °C.


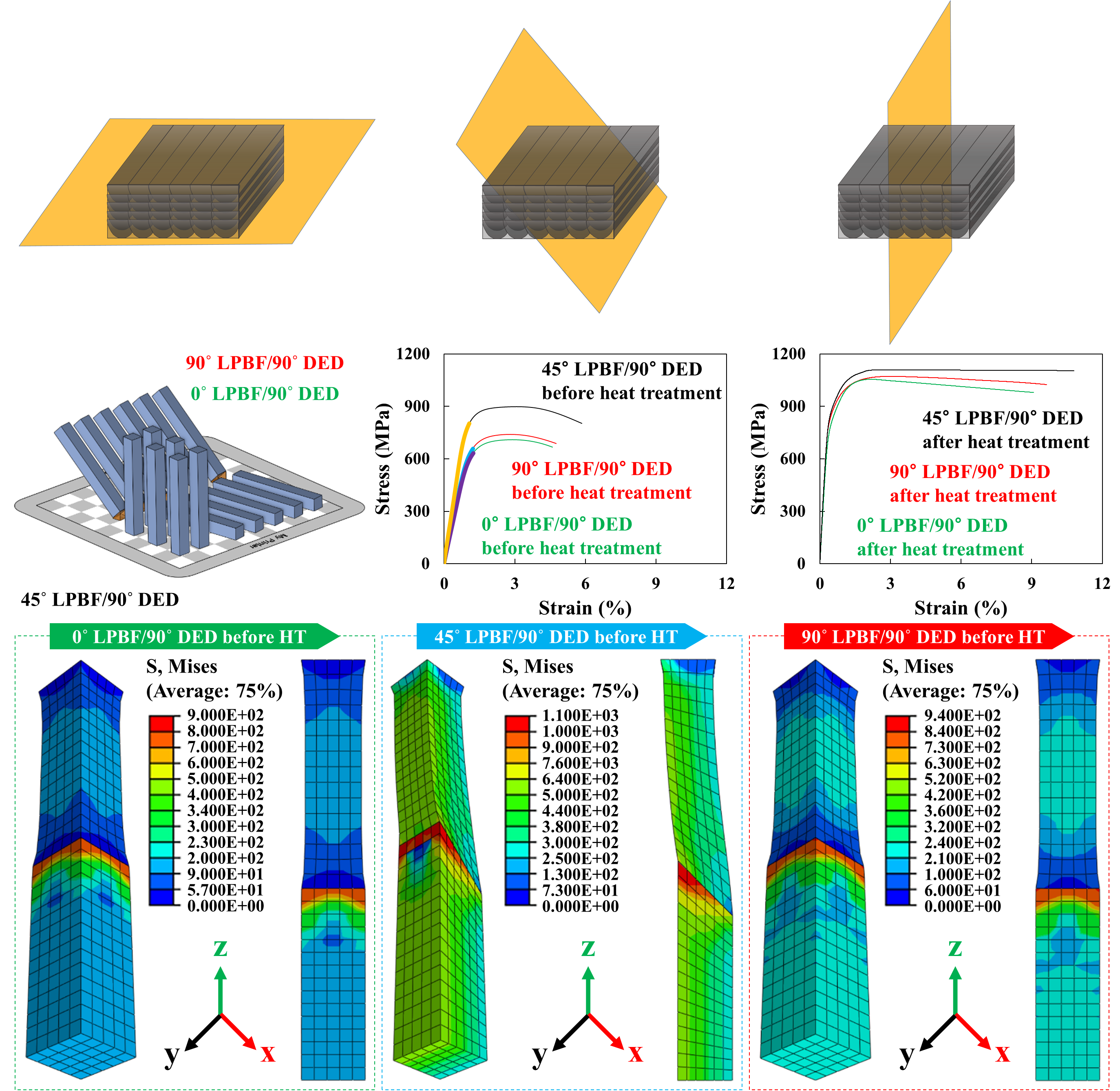


**Figure S8.** Schematic diagrams of the 3D-constructed melt pools built along the 0°, 45°, and 90° directions when using the LPBF process. When the BD of 45° is applied, a greater area of the plane passes through the center of each melt pool (thus leading to fewer microstructural defects and mechanical flaws) relative to the edge (which would lead to greater numbers of microstructural defects and mechanical flaws). Consequently, the sequential LPBF−DED structure built along the 45°/90° direction has a higher strength than those built along the 0°/90° and 90°/90° directions. The equivalent stress gradient distributions of the 48.1Al47.9Ti4.0V bimodal structures when additively manufactured by the sequential LPBF and DED processes with the BDs of 0°/90°, 45°/90°, and 90°/90° were simulated using FEM and compared to the experimentally measured TSs before HT. In the simulations, the presence of melt pools was ignored, although their different BDs were aligned definitely along the IBs of the bimodal structures. Thus, most of the external force and pressure applied to the bimodal structures were concentrated in the IBs. Comparatively, the external load on each region beyond the IBs of the bimodal structures was less concentrated owing to tight bonding between the γ- and γ′-like phases, in addition to the presence of V-based compounds.
